# Health risk assessment and metal contamination in fish, water and soil sediments in the East Kolkata Wetlands, India, Ramsar site

**DOI:** 10.1038/s41598-023-28801-y

**Published:** 2023-01-27

**Authors:** Neeraj Kumar, Nitish Kumar Chandan, Shashi Bhushan, Dilip Kumar Singh, Satish Kumar

**Affiliations:** 1 ICAR-National Institute of Abiotic Stress Management (NIASM), Malegaon, Baramati, Pune, 413115 India; 2 ICAR-Central Institute of Freshwater Aquaculture, Bhubaneshwar, 751002 India; 3 ICAR-Central Institute of Fisheries Education, Versova, Mumbai, 400061 India

**Keywords:** Environmental monitoring, Ichthyology

## Abstract

East Kolkata Wetlands (EKW) is an important site for fish culture in sewage-fed areas, which are major receivers of pollutants and wastages from Kolkata. EKW is internationally important as the Ramsar site was declared on Aug 2002 with an area of 125 km^2^. EKW is a natural water body where wastewater-fed natural aquaculture has been practiced for more than 70 years. It is ecologically vulnerable due to the discharge of toxic waste through sewage canals from cities. Assessing the EKW to understand the inflow and load of the toxic metal (s) in fish, water, and sediments samples is essential. The field (samples collection from 13 sites) and lab (determination of toxic level of metals) based research were carried out to assess metal toxicity and health risk assessment in EKW. The levels of eighteen metals (18), namely Chromium, Vanadium, Cobalt, Manganese, Copper, Nickel, Zinc, Silver, Molybdenum, Arsenic, Selenium, Tin, Gallium, Germanium, Strontium, Cadmium, Mercury, and Lead, were determined using Inductively coupled plasma mass spectrometry (ICP-MS) in five fish tissues viz. muscle, liver, kidney, gill and brain, along with the water samples and soil sediments in 13 sampling sites. The bioaccumulation and concentration of metals in fish tissues, soil sediments, and water samples were well within the safe level concerning the recommendation of different national and international agencies except for a few metals in a few sampling sites like Cd, As, and Pb. The geoaccumulation index (Igeo) was also determined in the soil sediments, indicating moderate arsenic, selenium, and mercury contamination in a few sites. The contamination index in water was also determined in 13 sampling sites. The estimated daily intake (EDI), reference dose (RfD), target hazard quotient (THQ), slope factor and cancer risk of Cr, Mn, Co, Ni, Cu, Zn, As, Se, Cd, Pb and Hg from fish muscle were determined. Based on the results of the present investigation, it is concluded that fish consumption in the East Kolkata Wetland (EKW) is safe. The effects of bioaccumulation of metals in muscle tissue were well within the safe level for consumption as recommended by WHO/FAO.

## Introduction

Metal pollution and its hazardous effect play a critical role in polluting aquatic systems leading to problems in aquatic animals and, subsequently, human health^1^. The contamination of the aquatic ecosystem with a wider range of contaminants and pollutants has due concern because of unplanned discharge, dumping of industrial effluents and different types of heavy metals released from industries, domestic and man-made activities over last few decades at global level^2–5^. However, identifying contaminants and pollutants and their prevention against dispersion is very much needed in this field condition. The organizations such as environmental protection agency (EPA), and agency for toxic substances and diseases registry (ATSDR) have reported that top 20 hazardous substances such as, arsenic (As), lead (Pb), and mercury (Hg) are the first, second and third position respectively, while cadmium (Cd) on 7th position. Therefore, heavy metals viz. chromium, manganese, cobalt, nickel, copper, zinc, molybdenum, silver, As, selenium, strontium, tin, cadmium, Pb and Hg are considered as most toxic to the human beings, animals, aquatic life, especially fish and their environment due to its non-biodegradable nature^3,6–8^. The metal contamination could result in be disturbance of ecological balance resulting in imbalance in diversity of aquatic ecosystems and build up in the food chain and finally responsible for adverse effects and death of aquatic organism^9–11^. The metal contamination in aquatic ecosystems is mainly due to natural processes like land erosion, weathering of rock deposits, and accelerated anthropogenic activities^12,13^. In the freshwater aquaculture systems, toxic metals are potentially accumulated in sediments, water, and aquatic organism and subsequently transferred to human beings through the food chain; however, a metal determination is mainly monitored in the water, sediments, and biota^14,15^. Whereas, low concentration was present in water and high concentration in sediments and biota such as fish^16,17^. Essential and non-essential metals are significant in ecotoxicology due to their highly persistent nature and are responsible for being toxic to living organisms^18–20^. The most of the previous studies focused on edible parts of the fish such as muscle including dispersion of metal in different parts of fish tissues such as the liver, kidneys, heart, gonads, bone, digestive tract, and brain^21–23^ and also in soil sediments and water. Heavy metals are an important group of chemical contaminants, and food and water serve as major source for their dispersal in the food chain. Industrial development enhanced the various kinds of discharge in the form of chemical effluents to the aquatic ecosystems, damaging the aquatic habitats and life of the animal, including fish. Metals discharged can damage the aquatic environment and species diversity due to their toxic nature and accumulative behavior^10,24–26^. There are different mechanisms for the uptake and elimination of heavy metals in aquatic organisms through various organs of the fish^6,27,28^.

India has the largest wetland in East Kolkata, named “East Kolkata Wetlands (EKW)” and or Sewage fed Fisheries and or Berries, covering around 12,000 ha area^29,30^, which was declared RAMSAR site on August 19, 2002. EKW has been functional since 1930, which is world's largest wastewater aquaculture, with an area of approximately 4000 h^29^ with an average fish production of 5 tons/hectare. Edwards and Pullin^31^ determined that East Kolkata Wetland/Sewage fed systems received more than 5,50,000 m^3^ untreated wastewater per day. The untreated wastewater contains many effluents obtained from 538 tanneries and 5500 industrial wastages^32^. This sewage-fed system has been treated with dilution and sedimentation of the solid particles, reducing the pollution level in the aquatic systems. As per the definition of the Ramsar Convention^33^, Wetlands are “areas of marsh, fern, peatland or water whether natural or artificial, permanent or temporary, with water, i.e. static or flowing, fresh, brackish or salt including areas of marine water, the depth of which at low tide does not exceed six meters.” The Ramsar Site Committee added “may incorporate riparian and coastal zones adjacent to the wetlands, and islands or bodies of marine water deeper than six meters at low tide lying within wetlands.” The first modern global inter-governmental treaty on wetlands conservation is known as “the Ramsar Convention”^33^.

Therefore, the present study is aimed to delineate the metals bioaccumulation in soil sediments, fish tissues (muscle, gill, liver, brain and kidney) and water samples to assess the degree of pollution using indices such as geo-chemical index (Igeo) and contamination index (CI), as well as addressing healthy and safety issues such as target hazard quotient (THQ) and cancer risk at East Kolkata Wetland.

## Materials and methods

### Ethics statement

The Research Advisory Committee approved the study protocol and the end-points of the experiments of ICAR-National Institute of Abiotic Stress Management (NIASM). All methods were carried out following relevant national and international guidelines and regulations. The study complies with the Animal Research: Reporting of in Vivo Experiments (ARRIVE) guidelines.

### Study area

**Figure 1:**
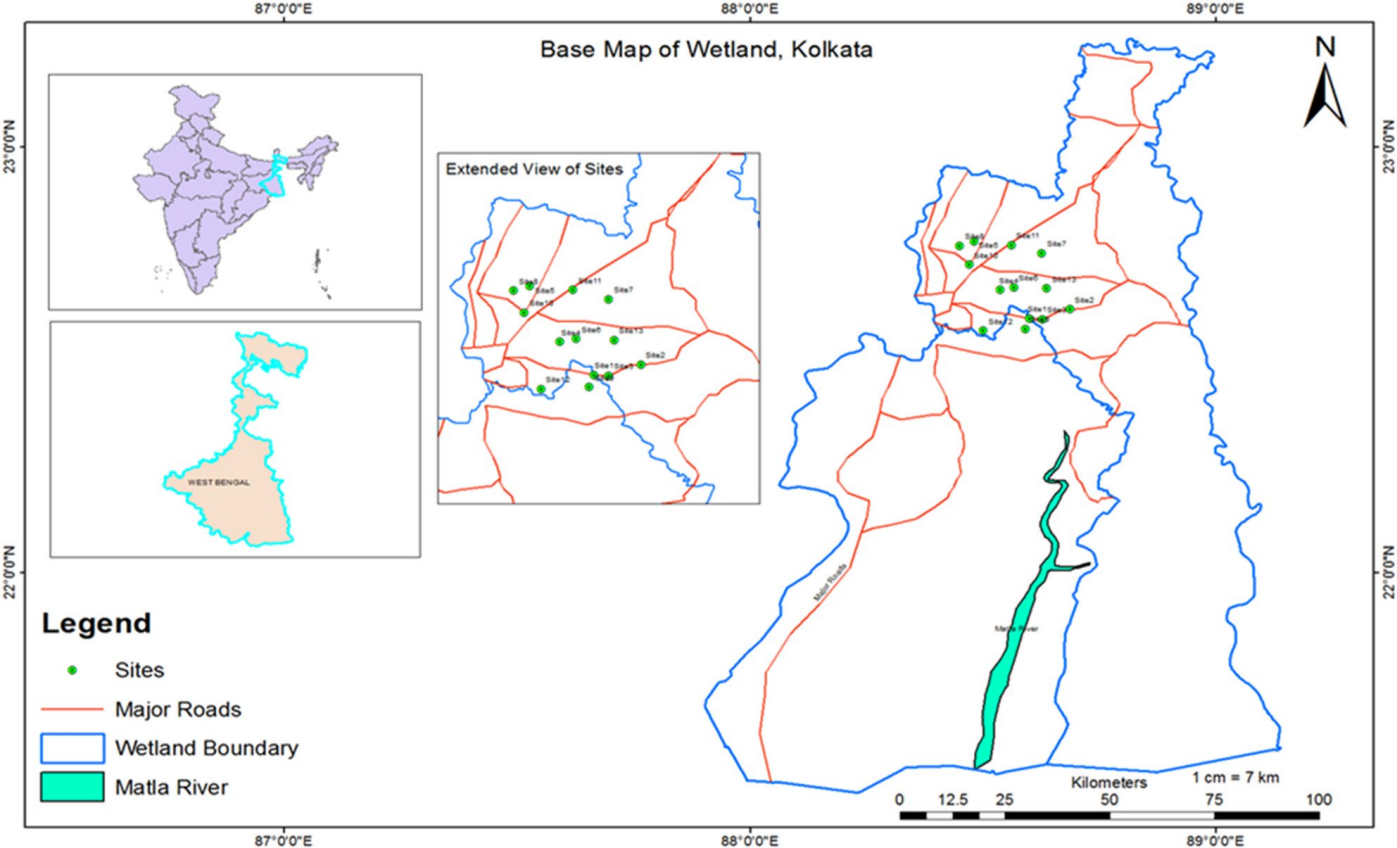
Base Map of 13 sampling sites of East Kolkata Wetland, Ramsar Site.

The investigation was conducted at East Kolkata Wetlands (EKW) system in June 2017 at 13 sampling sites and collected *Labeo rohita* for assessment of metals contamination and pollution index of EKW. The Ramsar sites are situated at Kulti Gong on the east side and the levee of the Hooghly River on the west side^29^ and are treated with a natural process such as sewage fed fish culture. It has unique and international status due to Ramsar (Ramsar site no. 1208) site which was declared on Aug 19, 2002^34^. The position of sampling sites has been recorded using the global positioning satellite system (GPS) using ArcGIS, esri, version 10.2.2 (Table S1 and Fig. 1).

### Sample collection

The soil sediments, water and fish were collected from 13 sampling sites of EKW. The water samples were collected from 5 cm of river depth in 250 ml sterile polyethylene plastic bottle in triplicate in each sampling site and finally pooled together. Soil sediment samples were also collected from the same sites where water was sampled and preserved in polythene bags. *Labeo rohita* of three individuals were collected from each sampling site, due to the most available and most acceptable fish in this region. The mean weight of fish was 365.68 ± 25 g. During sampling, we collected the different fish tissues (muscle, liver, gill, kidney and brain) at the sampling site and immediately stored them in the ice box. After arrival in the laboratory, samples were kept in a freezer at − 80 °C until further use.

### Sample preparation for metal analysis

During sampling, the water sample was collected and stored at 4 °C. The samples were filtered with 0.45 µm filter paper and mixed with 100 µl of pure HNO_3_ (69%, Himedia Laboratory Pvt. Ltd., Mumbai, India). The soil sediments were dried in sunlight and then kept in an oven at 65 °C. The dried soil samples were ground and passed through a 100 mesh sieve before they were analysed. For the heavy metal analysis, soil sediment (0.10 g) was digested with 5:1:1 mixture of HNO3: HCl: HF and made 50 ml volume with triple distilled water. The muscle, gill, liver, kidney, and brain of 0.5 g were collected during dissection and proceeded for acidic digestion in a microwave digestion system (Microwave Digestion System, Model START-D, SN-135177, Milestone, USA). The HNO_3_ and H_2_O_2_ were added in a 5:1 ratio for digestion of fish tissues and, after that, allowed to cool at room temperature, then filtered through Whatman paper with 0.45 mm pore size. After that, the solution was made up to 50 ml with double distilled water and proceeded for elements analysis through Inductively Coupled Plasma Mass Spectrometry (ICP-MS) (Agilent 7700 series, Agilent Technologies, USA). Metals such as vanadium (V), chromium (Cr), manganese (Mn), cobalt (Co), nickel (Ni), copper (Cu), zinc (Zn), molybdenum (Mo), silver (Ag), arsenic (As), selenium (Se), strontium (Sr), Tin (Sn), cadmium (Cd), lead (Pb) and mercury (Hg) were determined by ICP-MS. Multielement Calibration Standard (Agilent Technologies, USA) solutions of 10 μg/ml were used to prepare the calibration curve. The calibration curves with R^2^ > 0.999 were accepted for concentration calculation^6,27^.

### Environmental assessment (Degree of pollution) impact

#### Geoaccumulation index (Igeo)

To understand the current status of the environment and the heavy metal contamination with respect to the natural environment for the East Kolkata Wetland. The following equation applied for calculation of Geoaccumulation Index (Igeo) (Muller et al.^35^)

1 $${\text{Igeo}}\,{ = }\,{\text{Log}}_{{2}} \,\left[ {{\text{C}}_{{\text{n}}} /1.{5}\, \times \,{\text{B}}_{{\text{n}}} } \right],$$

where, C_n_ is the measured concentration of metal n in sediment, B_n_ is the geochemical background concentration of metal^36^ n and 1.5 is the background matrix correction factors due to lithogenic effects. As per Muller et al.^35^, classified as per Igeo such as If Igeo value is 0 represent unpolluted sediments, Igeo is 0–1 represent unpolluted to moderately polluted, Igeo 1–2, moderately polluted, Igeo 2–3, moderately to strongly polluted, Igeo 3–4, strongly polluted, Igeo 4–5 strongly to extremely polluted and Igeo more than 6 represents extremely polluted.

#### Contamination index (CI)

Contamination index is used for identification of enriched heavy metals with respect to the maximum admissible limit (MAL) standard as SON^37^; WHO^38^. The contamination index in water samples were calculated as.

$${\text{CI}}\, = \,\left[ {\left( {{\text{Concentration of the Total studied metals}}/{\text{ MAL of each metal}}} \right)/{\text{ total no}}.{\text{ of studied metal}}} \right]$$

2 $${\text{CI}}\, = \,\left[ {\left( {{\text{Cr}}/0.0{5} + {\text{Mn}}/0.{2}0 + {\text{Co}}/0.0{2} + {\text{Ni}}/0.0{2} + {\text{Cu}}/{1} + {\text{Zn}}/{3} + {\text{As}}/0.0{1} + {\text{Se}}/0.0{1} + {\text{Cd}}/0.00{3} + {\text{Hg}}/0.00{1} + {\text{ Pb}}/0.0{1}} \right)/11} \right].$$

The contamination index is classified as CI > 5 contaminated, CI 1–5 slight contaminated and CI < 1 represent not contaminated.

### Risk assessments

It is function of hazard which defined as the process of estimating the probability of occurrence of an event and the probable magnitude of adverse health effects on human exposures to environmental hazards over a specified time period^38^. The risk assessment includes hazard identification, exposure assessment, dose response (toxicity) and risk characterization^39^ and it is expressed in carcinogenic or a non-carcinogenic health risk such as slope factor represent carcinogen risk characterization and reference dose (RfD) for non-carcinogen characterization^40^.

#### Target hazard quotient (THQ)

3 $${\text{THQ}}\,:\,{\text{EDI}}\,/\,{\text{RfD,}}$$

Whereas

4 $${\text{EDI}}\, = \,\left[ {\left( {{\text{EF}} \times {\text{ED}} \times {\text{FIR}} \times {\text{C}}/{\text{W}} \times {\text{TA}}} \right)/{1} \times {1}0^{{ - {3}}} } \right],$$

Whereas EDI: Estimated daily intake (mg/kg/day), EF: Exposure frequency (365 days/year for people who eat fish), ED: Exposure duration (70 years, equivalent to average lifetime based on World Bank, 2012), FIR: Food ingestion rate (30 g/person/day for India)^29^, C: Metal concentration in sample, W: Average body weight of man (60 kg for Indian), TA: Average exposure time for non-carcinogenic (365 days × ED), RfD: Reference dose(mg/kg/day). RfD (mg/kg/day) for Cr 0.003, Mn 0.14, Co 0.10, Ni 0.02, Cu 0.04, Zn 0.3, As 0.0003, Se 0.005, Cd 0.001, Hg 0.004 and Pb 0.006 (US EPA IRIS, 2011; WHO, 2011). If THQ is exceeding 1.0, unacceptable risk and THQ is below 1.0, acceptable risk of non-carcinogenic effect on health^40^.

### Cancer risk (CR)

5 $${\text{Cancer Risk}}:{\text{ EDI }} \times {\text{ slope factor}}.$$

The slope factor (mg/kg/day) has been determined for few metals such as As 1.5 (US EPA IRIS,)^41^, Cd 0.0085 and Pb 6.3.

### Statistical analysis

Statistical Package statistically analyzed the data for the Social Sciences (SPSS) version 16. Data were subjected to one-way ANOVA (Analysis of variance) followed by duncan’s multiple range tests used to determine the significant differences between the means.

## Results

### Metal concentration in the water sample

The metal concentrations in the water sample collected from thirteen sampling sites are shown in Tables 1, 2. The concentration of metals in water sample were varies from 1.20–5.36, 1.17–1.89, 0.58–16.25, 0.27–0.68, 1.64–4.63, 2.12–5.05, 2.89–54.56, 0.05–0.23, 0.005–0.034, 0.09–0.18, 2.43–15.67, 2.04–8.82, 89.56–237.28, 0.02–0.30, 0.04–9.86, 0.27–4.69, 0.06–2.13 µg L^−1^ in V, Cr, Mn, Co, Ni, Cu, Zn, Mo, Ag, Ga, Ge, As, Se, Sr, Sn, Cd, Hg and Pb respectively.

**Table 1:** Concentrations (µg L^−1^) of V, Cr, Mn, Co, Ni, Cu, Zn, Mo and Ag in water sample collected from 13 different sites of East Kolkata Wetland.

Sites	V	Cr	Mn	Co	Ni	Cu	Zn	Mo	Ag
Site-1	3.07 ± 0.13	1.89 ± 0.05	16.25 ± 0.84	0.38 ± 0.04	4.28 ± 0.26	3.42 ± 0.08	5.26 ± 0.09	30.65 ± 0.39	0.16 ± 0.02
Site-2	5.36 ± 0.07	1.57 ± 0.03	1.50 ± 0.50	0.40 ± 0.01	3.33 ± 0.31	3.20 ± 0.10	5.44 ± 0.48	7.61 ± 0.19	0.05 ± 0.001
Site-3	3.77 ± 0.07	1.88 ± 0.05	3.22 ± 0.05	0.30 ± 0.04	4.63 ± 0.26	5.05 ± 0.30	54.56 ± 1.16	9.21 ± 0.32	0.10 ± 0.01
Site-4	4.04 ± 0.15	1.32 ± 0.10	5.75 ± 0.05	0.36 ± 0.01	3.50 ± 0.10	4.46 ± 0.19	30.86 ± 0.50	2.36 ± 0.14	0.14 ± 0.01
Site-5	2.74 ± 0.05	1.17 ± 0.05	3.89 ± 0.23	0.32 ± 0.02	4.34 ± 0.03	4.51 ± 0.12	14.33 ± 0.23	2.67 ± 0.15	0.13 ± 0.02
Site-6	2.54 ± 0.05	1.54 ± 0.02	0.64 ± 0.06	0.35 ± 0.02	4.08 ± 0.51	3.03 ± 0.12	4.97 ± 0.27	4.05 ± 0.08	0.05 ± 0.01
Site-7	2.49 ± 0.07	1.38 ± 0.03	0.58 ± 0.04	0.33 ± 0.04	2.91 ± 0.09	2.53 ± 0.09	4.20 ± 0.18	19.68 ± 0.16	0.05 ± 0.02
Site-8	1.61 ± 0.06	1.59 ± 0.01	1.20 ± 0.04	0.37 ± 0.04	4.05 ± 0.15	2.30 ± 0.15	3.33 ± 0.31	1.89 ± 0.16	0.05 ± 0.01
Site-9	1.20 ± 0.01	1.27 ± 0.15	1.65 ± 0.14	0.41 ± 0.02	2.48 ± 0.14	3.42 ± 0.12	2.89 ± 0.04	2.68 ± 0.26	0.23 ± 0.02
Site-10	1.42 ± 0.02	1.65 ± 0.17	1.48 ± 0.03	0.61 ± 0.01	1.64 ± 0.16	2.78 ± 0.31	4.65 ± 0.15	3.74 ± 0.34	0.17 ± 0.003
Site-11	1.56 ± 0.12	1.35 ± 0.13	1.62 ± 0.04	0.27 ± 0.03	3.45 ± 0.24	2.12 ± 0.27	11.32 ± 0.53	4.72 ± 0.17	0.13 ± 0.004
Site-12	1.45 ± 0.31	1.78 ± 0.24	1.48 ± 0.18	0.36 ± 0.04	1.78 ± 0.31	3.41 ± 0.34	2.56 ± 0.42	1.89 ± 0.03	0.16 ± 0.006
Site-13	1.98 ± 0.41	1.62 ± 0.18	1.92 ± 0.16	0.68 ± 0.03	2.49 ± 0.04	2.47 ± 0.05	4.65 ± 0.034	6.45 ± 0.17	0.12 ± 0.042

Data expressed as mean ± SE (n = 6).

**Table 2:** Concentrations (µg L^−1^) of Ga, Ge, Se, Sr, Sn, Cd, Hg and Pb in in water sample collected from 13 different sites of East Kolkata Wetland.

Sites	Ga	Ge	As	Se	Sr	Sn	Cd	Hg	Pb
Site-1	0.026 ± 0.01	0.10 ± 0.06	4.56 ± 0.33	2.04 ± 0.46	237.28 ± 6.86	0.26 ± 0.01	0.12 ± 0.02	4.69 ± 0.21	0.19 ± 0.001
Site-2	0.008 ± 0.001	0.13 ± 0.03	6.81 ± 0.24	6.39 ± 0.26	217.81 ± 5.24	0.09 ± 0.001	0.04 ± 0.02	0.79 ± 0.12	0.20 ± 0.04
Site-3	0.023 ± 0.001	0.16 ± 0.03	3.96 ± 0.23	6.90 ± 1.38	226.60 ± 6.10	0.16 ± 0.01	9.86 ± 0.03	1.51 ± 0.25	0.83 ± 0.02
Site-4	0.015 ± 0.002	0.13 ± 0.02	6.71 ± 0.43	8.82 ± 1.15	229.08 ± 6.23	0.30 ± 0.003	0.40 ± 0.05	0.31 ± 0.12	2.13 ± 0.11
Site-5	0.008 ± 0.001	0.10 ± 0.01	15.67 ± 0.72	5.37 ± 1.23	99.89 ± 2.97	0.09 ± 0.001	0.18 ± 0.03	0.27 ± 0.09	0.61 ± 0.01
Site-6	0.005 ± 0.001	0.09 ± 0.01	6.10 ± 0.25	3.96 ± 0.34	135.50 ± 2.90	0.17 ± 0.003	0.07 ± 0.02	0.34 ± 0.03	0.14 ± 0.03
Site-7	0.015 ± 0.001	0.14 ± 0.02	4.19 ± 0.29	5.37 ± 0.59	189.22 ± 2.60	0.13 ± 0.001	0.55 ± 0.11	1.16 ± 0.18	0.06 ± 0.001
Site-8	0.010 ± 0.001	0.17 ± 0.03	3.22 ± 0.34	8.31 ± 1.09	128.69 ± 2.0	0.07 ± 0.002	0.55 ± 0.11	0.31 ± 0.06	0.09 ± 0.001
Site-9	0.002 ± 0.003	0.12 ± 0.04	2.56 ± 0.18	4.68 ± 0.65	89.56 ± 0.45	0.02 ± 0.004	0.27 ± 0.002	0.38 ± 0.03	0.14 ± 0.003
Site-10	0.012 ± 0.001	0.17 ± 0.003	3.15 ± 0.17	5.78 ± 0.34	111.27 ± 1.56	0.17 ± 0.003	0.34 ± 0.001	0.75 ± 0.04	0.17 ± 0.004
Site-11	0.034 ± 0.002	0.11 ± 0.03	4.62 ± 0.26	4.68 ± 0.14	119.23 ± 2.45	0.05 ± 0.004	0.31 ± 0.004	0.91 ± 0.06	0.23 ± 0.002
Site-12	0.027 ± 0.003	0.13 ± 0.01	2.43 ± 0.34	6.45 ± 0.37	107.56 ± 3.21	0.13 ± 0.03	0.46 ± 0.003	0.82 ± 0.02	0.12 ± 0.005
Site-13	0.026 ± 0.004	0.18 ± 0.002	2.75 ± 0.17	2.41 ± 0.47	121.53 ± 4.56	0.21 ± 0.005	0.78 ± 0.001	0.76 ± 0.03	0.24 ± 0.003

Data expressed as mean ± SE (n = 6).

### Metal concentration in soil sediments sample

The soil sediment samples collected from thirteen sampling sites from East Kolkata Wetland are presented in Tables 3, 4. The average metals concentration of V, Cr, Mn, Co, Ni, Cu, Zn, Mo, Ag, Ga, Ge, As, Se, Sr, Sn, Cd, Hg and Pb followed as 8.41, 7.10, 63.23, 1.51, 4.53, 7.12, 28.87, 0.27, 0.10, 2.43, 0.55, 5.79, 1.29, 6.28, 1.85, 0.42, 0.23 and 4.32 mg kg^−1^ respectively in each site. The most toxic trace metals such as As, Cd, Hg, and Pb were highest at sites 13 (7.65), 3 (0.75), 13 (0.78) and 3 (10.60 mg kg^−1^ soil) respectively.

**Table 3:** Concentrations (mg kg^−1^) of V, Cr, Mn, Co, Ni, Cu, Zn, Mo and Ag in soil sediments sample collected from 13 different sites of East Kolkata Wetland.

Sites	V	Cr	Mn	Co	Ni	Cu	Zn	Mo	Ag
Site-1	10.98 ± 0.69	8.15 ± 0.31	84.50 ± 3.57	1.91 ± 0.07	5.22 ± 0.15	6.71 ± 0.31	26.30 ± 8.89	0.37 ± 0.051	0.10 ± 0.04
Site-2	9.80 ± 0.78	9.92 ± 0.84	66.97 ± 4.87	1.71 ± 0.14	5.40 ± 0.79	8.11 ± 0.60	26.61 ± 2.60	0.14 ± 0.07	0.14 ± 0.04
Site-3	9.50 ± 0.59	8.69 ± 1.03	63.74 ± 7.59	1.68 ± 0.29	5.10 ± 0.71	11.97 ± 1.03	55.00 ± 4.97	0.22 ± 0.10	0.22 ± 0.02
Site-4	10.59 ± 1.16	8.37 ± 0.32	71.98 ± 6.56	1.76 ± 0.31	5.31 ± 0.63	7.10 ± 0.77	25.36 ± 2.04	0.12 ± 0.02	0.10 ± 0.06
Site-5	8.42 ± 0.98	6.26 ± 1.10	47.99 ± 2.07	1.06 ± 0.04	4.34 ± 0.41	5.43 ± 0.39	18.93 ± 1.89	0.18 ± 0.02	0.06 ± 0.08
Site-6	7.68 ± 0.57	6.61 ± 0.90	52.52 ± 5.08	1.44 ± 0.15	4.00 ± 0.59	5.48 ± 1.05	19.51 ± 1.03	0.10 ± 0.007	0.08 ± 0.02
Site-7	8.44 ± 0.85	7.59 ± 0.32	59.76 ± 3.58	1.57 ± 0.11	4.52 ± 0.45	6.71 ± 0.34	21.86 ± 2.78	0.11 ± 0.004	0.09 ± 0.05
Site-8	4.65 ± 0.26	4.87 ± 0.36	52.45 ± 4.58	1.24 ± 0.31	3.48 ± 0.49	4.65 ± 0.45	17.56 ± 1.65	0.24 ± 0.014	0.05 ± 0.01
Site-9	6.78 ± 0.34	6.45 ± 0.75	61.85 ± 4.56	1.35 ± 0.41	4.62 ± 0.16	8.89 ± 0.67	24.56 ± 2.78	0.31 ± 0.016	0.17 ± 0.001
Site-10	8.49 ± 1.56	6.41 ± 0.43	72.56 ± 3.78	1.45 ± 0.45	4.17 ± 0.34	7.65 ± 1.23	23.47 ± 1.56	0.48 ± 0.01	0.01 ± 0.002
Site-11	6.72 ± 0.61	8.75 ± 0.27	71.59 ± 8.56	1.61 ± 0.31	4.23 ± 0.47	8.12 ± 1.78	34.56 ± 1.91	0.33 ± 0.021	0.06 ± 0.003
Site-12	9.45 ± 0.34	2.89 ± 0.18	43.51 ± 2.45	1.75 ± 0.18	4.18 ± 0.62	4.93 ± 1.31	32.98 ± 4.56	0.45 ± 0.03	0.14 ± 0.02
Site-13	7.89 ± 1.56	7.31 ± 0.62	72.54 ± 2.46	1.05 ± 0.27	4.27 ± 0.37	6.75 ± 1.78	48.63 ± 3.45	0.51 ± 0.02	0.12 ± 0.01

Data expressed as mean ± SE (n = 6).

**Table 4:** Concentrations (mg kg^−1^) of Ga, Ge, Se, Sr, Sn, Cd, Hg and Pb in soil sediments sample collected from 13 different sites of East Kolkata Wetland.

Sites	Ga	Ge	As	Se	Sr	Sn	Cd	Hg	Pb
Site-1	2.53 ± 0.01	0.75 ± 0.13	6.11 ± 1.29	2.94 ± 0.56	7.18 ± 0.37	1.76 ± 0.17	0.70 ± 0.03	0.16 ± 0.06	2.94 ± 0.26
Site-2	2.51 ± 0.57	0.62 ± 0.25	5.40 ± 1.12	0.95 ± 0.04	6.36 ± 0.70	1.56 ± 0.35	0.41 ± 0.12	0.16 ± 0.02	3.46 ± 0.54
Site-3	2.43 ± 0.40	0.59 ± 0.16	5.09 ± 1.76	1.19 ± 0.63	8.07 ± 0.51	2.22 ± 0.33	0.75 ± 0.09	0.26 ± 0.12	10.60 ± 1.34
Site-4	2.71 ± 0.28	0.64 ± 0.05	5.10 ± 1.19	0.96 ± 0.41	6.00 ± 0.64	1.68 ± 0.24	0.10 ± 0.09	0.14 ± 0.03	4.00 ± 0.39
Site-5	1.94 ± 0.57	0.46 ± 0.03	5.02 ± 0.90	0.61 ± 0.14	3.60 ± 0.27	2.22 ± 0.12	0.11 ± 0.06	0.05 ± 0.001	2.49 ± 0.29
Site-6	2.03 ± 0.17	0.51 ± 0.11	5.12 ± 2.01	0.75 ± 0.08	7.29 ± 0.68	1.58 ± 0.26	0.20 ± 0.12	0.09 ± 0.003	2.80 ± 0.34
Site-7	2.20 ± 0.15	0.49 ± 0.03	4.41 ± 0.56	0.87 ± 0.02	7.41 ± 0.59	3.61 ± 0.07	0.28 ± 0.13	0.10 ± 0.001	2.97 ± 0.43
Site-8	2.04 ± 0.09	0.47 ± 0.02	6.45 ± 0.14	1.45 ± 0.45	5.47 ± 0.31	1.45 ± 0.42	0.21 ± 0.004	0.13 ± 0.004	3.56 ± 0.18
Site-9	2.19 ± 0.31	0.46 ± 0.12	6.78 ± 0.21	1.68 ± 0.41	4.68 ± 0.34	2.01 ± 0.26	0.35 ± 0.013	0.05 ± 0.006	4.79 ± 0.34
Site-10	2.34 ± 0.04	0.58 ± 0.04	6.45 ± 1.36	1.75 ± 0.34	5.21 ± 0.52	1.62 ± 0.31	0.74 ± 0.02	0.27 ± 0.01	5.19 ± 0.42
Site-11	2.65 ± 0.03	0.57 ± 0.03	6.86 ± 0.18	1.62 ± 0.18	6.17 ± 0.42	1.73 ± 0.24	0.31 ± 0.03	0.36 ± 0.003	1.75 ± 0.31
Site-12	2.78 ± 0.04	0.64 ± 0.04	4.89 ± 0.65	0.87 ± 0.31	6.37 ± 0.37	1.49 ± 0.016	0.52 ± 0.02	0.45 ± 0.01	4.92 ± 0.28
Site-13	3.21 ± 0.02	0.37 ± 0.06	7.65 ± 0.78	1.08 ± 0.17	7.89 ± 0.18	1.06 ± 0.04	0.74 ± 0.04	0.78 ± 0.03	6.73 ± 0.47

Data expressed as mean ± SE (n = 6).

### Metals concentration in fish tissues

The muscle, gill, kidney, liver, and brain tissues of *L. rohita* collected from thirteen sampling sites of East Kolkata Wetland (EKW) are shown in Tables 5, 6, 7, 8, 9, 10, 11, 12, 13 and 14. The average concentration of V, Cr, Mn, Co, Ni, Cu, Zn, Mo, Ag, Ga, Ge, As, Se, Sr, Sn, Cd, Hg and Pb followed as 0.17, 1.60, 0.49, 0.03, 1.04, 5.75, 9.96, 0.32, 0.06, 0.19, 0.01, 0.01, 8.76, 0.73, 11.72, 6.75, 0.11 and 0.37 mg kg^−1^ respectively in muscle tissue. The highest concentration of Cr (2.48 mg kg^−1^), Mn (1.08 mg kg^−1^), Co (0.055 mg kg^−1^), Ni (2.09 mg kg^−1^), Cu (6.83 mg kg^−1^), Zn (24.26 mg kg^−1^), Ag (2.08 mg kg^−1^), As (0.81 mg kg^−1^), Se (1.21 mg kg^−1^), Sr (3.82 mg kg^−1^) Sn (21.28 mg kg^−1^), Cd (0.95 mg kg^−1^), Hg (0.25 mg kg^−1^) and Pb (1.23 mg kg^−1^ diet) were observed at site 11, 10, 9, 2, 2, 13, 11, 4, 12, 9, 3, 2, 1, and 2 respectively in muscle tissue (Tables 5, 6). Similarly, Cr (4.59 mg kg^−1^), Mn (3.87 mg kg^−1^), Ni (1.33 mg kg^−1^), Cu (4.14 mg kg^−1^), Zn (34.14 mg kg^−1^), Ag (0.31 mg kg^−1^), As (1.21 mg kg^−1^), Se (13.73 mg kg^−1^), Sr (22.10 mg kg^−1^) Sn (12.98 mg kg^−1^), Cd (3.42 mg kg^−1^), Hg (0.18 mg kg^−1^) and Pb (0.29 mg kg^−1^) were observed at site 9, 6, 9, 1, 7, 6, 11, 1, 4, 7, 1, 8 and 4 respectively in the gill tissue (Tables 7, 8). Moreover, the concentration of V, Cr, Mn, Co, Ni, Cu, Zn, Mo, and Ag in kidney was depicted in Table 9, whereas Ga, Ge, As, Se, Sr, Sn, Cd, Hg y, and Pb concentration in kidney was mentioned in the Table 10. The most toxic metals, such as As, Cd, Hg, Pb, Cr, Ni, and Cu, were at the highest level at sites 1, 10, 1, 2, 1, 1, and 9 in kidney tissue, respectively (Tables 9, 10). Moreover, liver tissue metals were higher at sites 1, 4, and 10. The As (0.20 mg kg^−1^) concentration was higher at site 13 than at all other sites. In the case of Cd (0.13 mg kg^−1^), site 2 and 13, was high, whereas Hg (0.13 mg kg^−1^) at site 12 and Pb (0.34 mg kg^−1^) at site 1 and 2. Cr, Mn, Ni, and Cu were higher at sites 1, 2, 6, and 9 in the liver tissue (Tables 11, 12). The metals bioaccumulation in the brain tissue was followed at sites 2 and 3 the As (0.15 mg kg^−1^) was the highest. Whereas, sites 11 and 1, Hg and Pb were higher, and sites 7, 3, 2, 11, 13 Cr, Mn, Ni, Cu, and Zn were higher in comparison to all other sites (Tables 13, 14). The metal bioaccumulation in all the sites was well within the safe level recommended by different National and International agencies except for a few sites. The average value of metals in V, Cr, Mn, Co, Ni, Cu, Zn, Mo, Ag, Ga, Ge, As, Se, Sr, Sn, Cd, Hg and Pb in gill, kidney, liver and brain tissues of *L. rohita* followed as 0.09, 1.03, 2.14, 0.06, 0.92, 3.75, 21.24, 0.06, 0.06, 0.01, 0.01, 0.22, 3.17, 12.17, 7.76, 0.55, 0.06, 0.22 mg kg^−1^ and 0.08, 0.70, 0.59, 0.06, 0.74, 4.43, 17.36, 0.09, 0.25, 0.01, 0.02, 0.12, 0.41, 0.34, 8, 0.54, 0.02, 0.26 and 0.09, 0.52, 0.93, 0.03, 0.82, 5.86, 20, 0.14, 0.19, 0.01, 0.06, 0.16, 4.66, 0.29, 4.13, 0.09, 0.03, 0.25 and 0.06, 0.43, 0.42, 0.02, 0.63, 3.21, 11.04, 0.06, 0.02, 0.01, 0.02, 0.10, 0.36, 0.63, 7.48, 0.03, 0.06, 0.17 mg kg^−1^ respectively in metals, and tissues.

**Table 5:** Concentrations (mg kg^-1^) of V, Cr, Mn, Co, Ni, Cu, Zn, Mo and Ag in muscle tissue of *Labeo rohita* collected from 13 different sites of East Kolkata Wetland.

Sites	V	Cr	Mn	Co	Ni	Cu	Zn	Mo	Ag
Site-1	0.24 ± 0.04	0.74 ± 0.04	0.69 ± 0.09	0.055 ± 0.03	0.79 ± 0.12	6.13 ± 0.35	9.73 ± 0.26	0.12 ± 0.013	0.34 ± 0.03
Site-2	0.18 ± 0.04	1.72 ± 0.32	0.39 ± 0.09	0.025 ± 0.02	2.09 ± 0.45	6.83 ± 1.41	8.29 ± 2.79	0.08 ± 0.001	0.29 ± 0.03
Site-3	0.15 ± 0.06	2.10 ± 0.25	0.29 ± 0.07	0.019 ± 0.02	1.20 ± 0.21	6.01 ± 0.40	7.74 ± 0.91	0.07 ± 0.006	2.88 ± 0.29
Site-4	0.17 ± 0.05	2.41 ± 0.43	0.30 ± 0.02	0.017 ± 0.01	1.40 ± 0.11	6.47 ± 0.63	7.46 ± 0.90	0.05 ± 0.001	0.54 ± 0.05
Site-5	0.19 ± 0.05	2.05 ± 0.14	0.45 ± 0.01	0.026 ± 0.02	0.60 ± 0.17	6.31 ± 1.16	7.75 ± 1.02	0.07 ± 0.004	0.26 ± 0.03
Site-6	0.18 ± 0.05	1.73 ± 0.36	0.31 ± 0.04	0.017 ± 0.01	1.31 ± 0.28	6.13 ± 0.35	8.11 ± 0.98	0.09 ± 0.003	0.13 ± 0.01
Site-7	0.20 ± 0.09	1.56 ± 0.16	0.31 ± 0.02	0.021 ± 0.02	0.61 ± 0.23	6.48 ± 0.81	9.55 ± 0.97	0.05 ± 0.005	0.11 ± 0.01
Site-8	0.18 ± 0.02	1.84 ± 0.14	0.26 ± 0.05	0.034 ± 0.02	1.53 ± 0.47	5.87 ± 0.56	8.30 ± 0.83	0.05 ± 0.003	0.24 ± 0.02
Site-9	0.29 ± 0.07	1.09 ± 0.21	0.86 ± 0.18	0.055 ± 0.04	0.59 ± 0.11	6.12 ± 0.54	9.58 ± 0.71	0.07 ± 0.003	0.55 ± 0.06
Site-10	0.27 ± 0.05	1.41 ± 0.21	1.08 ± 0.21	0.042 ± 0.02	0.58 ± 0.02	6.50 ± 0.66	8.88 ± 0.47	0.03 ± 0.004	0.08 ± 0.01
Site-11	0.06 ± 0.006	2.48 ± 0.10	0.39 ± 0.05	0.020 ± 0.03	1.07 ± 0.29	3.90 ± 0.73	7.77 ± 1.53	0.06 ± 0.007	2.08 ± 0.21
Site-12	0.09 ± 0.005	0.97 ± 0.13	0.55 ± 0.05	0.026 ± 0.01	0.87 ± 0.31	4.00 ± 0.28	12.04 ± 1.03	0.08 ± 0.01	17.10 ± 1.71
Site-13	0.06 ± 0.003	0.63 ± 0.07	0.47 ± 0.08	0.019 ± 0.02	0.92 ± 0.02	3.93 ± 0.22	24.26 ± 0.83	0.04 ± 0.001	0.21 ± 0.02

Data expressed as mean ± SE (n = 6).

**Table 6:** Concentrations (mg kg^−1^) of Ga, Ge, Se, Sr, Sn, Cd, Hg and Pb in muscle tissue of *Labeo rohita* collected from 13 different sites of East Kolkata Wetland.

Sites	Ga	Ge	As	Se	Sr	Sn	Cd	Hg	Pb
Site-1	0.023 ± 0.005	0.015 ± 0.003	0.30 ± 0.14	0.37 ± 0.03	2.50 ± 0.39	9.15 ± 0.17	0.86 ± 0.04	0.257 ± 0.06	0.30 ± 0.04
Site-2	0.007 ± 0.001	0.005 ± 0.002	0.35 ± 0.07	0.23 ± 0.04	0.18 ± 0.03	14.31 ± 2.44	49.07 ± 13.97	0.153 ± 0.001	1.23 ± 0.23
Site-3	0.005 ± 0.001	0.008 ± 0.002	0.29 ± 0.01	0.28 ± 0.03	0.12 ± 0.03	21.28 ± 1.88	0.75 ± 0.29	0.063 ± 0.21	0.32 ± 0.09
Site-4	0.010 ± 0.003	0.010 ± 0.002	0.81 ± 0.32	0.47 ± 0.03	0.32 ± 0.10	15.10 ± 0.72	0.48 ± 0.08	0.074 ± 0.03	0.23 ± 0.04
Site-5	0.006 ± 0.002	0.010 ± 0.001	0.21 ± 0.23	0.33 ± 0.10	0.29 ± 0.06	8.81 ± 1.21	0.05 ± 0.06	0.134 ± 0.13	0.45 ± 0.07
Site-6	0.006 ± 0.001	0.006 ± 0.002	0.27 ± 0.24	0.40 ± 0.07	0.13 ± 0.04	14.49 ± 1.08	0.14 ± 0.01	0.249 ± 0.27	0.20 ± 0.06
Site-7	0.009 ± 0.001	0.010 ± 0.003	0.33 ± 0.17	0.25 ± 0.02	0.17 ± 0.03	9.36 ± 0.68	0.11 ± 0.01	0.060 ± 0.01	0.26 ± 0.01
Site-8	0.007 ± 0.001	0.009 ± 0.002	0.27 ± 0.21	0.55 ± 0.01	0.16 ± 0.01	13.62 ± 1.00	0.12 ± 0.01	0.093 ± 0.01	0.37 ± 0.02
Site-9	0.010 ± 0.002	0.009 ± 0.003	0.36 ± 0.33	0.39 ± 0.02	3.82 ± 0.39	9.14 ± 0.60	35.71 ± 4.00	0.104 ± 0.32	0.51 ± 0.10
Site-10	0.007 ± 0.001	0.010 ± 0.002	0.30 ± 0.07	0.74 ± 0.12	0.90 ± 0.10	8.77 ± 0.86	0.40 ± 0.16	0.063 ± 0.01	0.33 ± 0.05
Site-11	0.006 ± 0.001	0.005 ± 0.001	0.20 ± 0.02	0.49 ± 1.91	0.38 ± 0.07	14.14 ± 1.38	0.05 ± 0.001	0.045 ± 0.001	0.23 ± 0.04
Site-12	0.007 ± 0.002	0.010 ± 0.002	0.19 ± 0.01	0.33 ± 1.33	0.24 ± 0.06	6.83 ± 0.42	0.05 ± 0.001	0.060 ± 0.01	0.25 ± 0.01
Site-13	0.006 ± 0.001	0.015 ± 0.002	0.21 ± 0.11	1.21 ± 0.06	0.24 ± 0.04	7.36 ± 0.94	0.03 ± 0.001	0.052 ± 0.01	0.16 ± 0.01

Data expressed as mean ± SE (n = 6).

**Table 7:** Concentrations (mg kg^−1^) of V, Cr, Mn, Co, Ni, Cu, Zn, Mo and Ag in gill tissue of *Labeo rohita* collected from 13 different sites of East Kolkata Wetland.

Sites	V	Cr	Mn	Co	Ni	Cu	Zn	Mo	Ag
Site-1	0.10 ± 0.03	0.83 ± 0.06	1.82 ± 0.03	0.06 ± 0.003	0.77 ± 0.29	4.14 ± 0.07	10.64 ± 0.61	0.06 ± 0.004	0.02 ± 0.002
Site-2	0.06 ± 0.003	0.73 ± 0.08	1.47 ± 0.17	0.05 ± 0.005	0.94 ± 0.05	3.81 ± 0.49	13.34 ± 0.27	0.04 ± 0.003	0.02 ± 0.002
Site-3	0.10 ± 0.04	0.52 ± 0.11	1.95 ± 0.13	0.04 ± 0.002	0.89 ± 0.17	3.87 ± 0.39	12.13 ± 2.34	0.04 ± 0.002	0.01 ± 0.002
Site-4	0.13 ± 0.05	0.71 ± 0.05	3.00 ± 0.28	0.06 ± 0.004	1.07 ± 0.56	4.00 ± 0.74	20.28 ± 0.51	0.08 ± 0.004	0.03 ± 0.004
Site-5	0.12 ± 0.03	0.62 ± 0.21	1.88 ± 0.09	0.06 ± 0.003	1.14 ± 0.32	4.03 ± 0.24	21.71 ± 3.96	0.05 ± 0.007	0.01 ± 0.001
Site-6	0.07 ± 0.05	0.73 ± 0.21	3.87 ± 0.52	0.07 ± 0.001	0.73 ± 0.13	4.03 ± 0.74	26.47 ± 2.76	0.05 ± 0.006	0.31 ± 0.06
Site-7	0.06 ± 0.02	1.22 ± 0.20	0.75 ± 0.18	0.04 ± 0.001	1.23 ± 0.40	4.05 ± 0.58	34.14 ± 4.73	0.05 ± 0.007	0.03 ± 0.001
Site-8	0.07 ± 0.04	1.01 ± 0.15	1.56 ± 0.25	0.06 ± 0.004	0.87 ± 0.04	3.54 ± 0.75	15.19 ± 1.00	0.07 ± 0.006	0.02 ± 0.01
Site-9	0.13 ± 0.03	4.59 ± 0.66	2.37 ± 0.46	0.08 ± 0.003	1.33 ± 0.44	3.89 ± 0.52	23.62 ± 2.46	0.06 ± 0.001	0.04 ± 0.01
Site-10	0.09 ± 0.01	0.59 ± 0.10	2.62 ± 0.42	0.05 ± 0.002	0.73 ± 0.02	3.36 ± 0.23	29.35 ± 3.69	0.08 ± 0.003	0.16 ± 0.08
Site-11	0.09 ± 0.07	0.61 ± 0.15	2.81 ± 0.39	0.05 ± 0.002	0.67 ± 0.18	3.48 ± 0.16	28.44 ± 3.53	0.09 ± 0.006	0.07 ± 0.001
Site-12	0.07 ± 0.03	0.61 ± 0.15	2.21 ± 0.50	0.05 ± 0.002	0.61 ± 0.18	3.17 ± 0.48	21.14 ± 4.26	0.08 ± 0.003	0.10 ± 0.07
Site-13	0.08 ± 0.05	0.59 ± 0.08	1.51 ± 0.14	0.05 ± 0.001	0.99 ± 0.20	3.39 ± 0.70	19.73 ± 2.31	0.08 ± 0.009	0.01 ± 0.001

Data expressed as mean ± SE (n = 6).

**Table 8:** Concentrations (mg kg^−1^) of Ga, Ge, Se, Sr, Sn, Cd, Hg and Pb in gill tissue of *Labeo rohita* collected from 13 different sites of East Kolkata Wetland.

Sites	Ga	Ge	As	Se	Sr	Sn	Cd	Hg	Pb
Site-1	0.011 ± 0.002	0.019 ± 0.002	0.17 ± 0.02	13.73 ± 3.70	8.79 ± 0.93	6.91 ± 1.06	3.42 ± 0.40	0.01 ± 0.004	0.20 ± 0.03
Site-2	0.008 ± 0.001	0.012 ± 0.001	0.16 ± 0.01	10.85 ± 1.54	7.68 ± 0.40	7.13 ± 0.37	0.12 ± 0.12	0.03 ± 0.015	0.27 ± 0.06
Site-3	0.007 ± 0.002	0.012 ± 0.001	0.11 ± 0.05	1.36 ± 0.03	5.33 ± 1.01	7.61 ± 1.16	0.09 ± 0.001	0.07 ± 0.019	0.17 ± 0.05
Site-4	0.015 ± 0.002	0.013 ± 0.001	0.10 ± 0.04	5.83 ± 1.01	22.10 ± 2.14	7.18 ± 1.32	0.05 ± 0.003	0.02 ± 0.017	0.29 ± 0.03
Site-5	0.009 ± 0.002	0.009 ± 0.001	0.10 ± 0.01	4.82 ± 1.42	18.24 ± 2.16	6.80 ± 0.52	3.06 ± 0.31	0.02 ± 0.013	0.26 ± 0.10
Site-6	0.014 ± 0.003	0.019 ± 0.002	0.11 ± 0.03	0.69 ± 0.17	19.49 ± 2.50	6.84 ± 0.53	0.11 ± 0.01	0.16 ± 0.010	0.23 ± 0.03
Site-7	0.006 ± 0.002	0.012 ± 0.001	0.24 ± 0.02	1.41 ± 0.77	6.38 ± 0.79	12.98 ± 1.30	0.04 ± 0.001	0.04 ± 0.013	0.21 ± 0.06
Site-8	0.010 ± 0.002	0.014 ± 0.001	0.10 ± 0.05	0.12 ± 0.01	10.62 ± 1.70	7.95 ± 1.00	0.04 ± 0.001	0.18 ± 0.021	0.18 ± 0.05
Site-9	0.016 ± 0.003	0.015 ± 0.002	0.11 ± 0.01	1.56 ± 0.27	21.04 ± 2.94	7.36 ± 1.11	0.09 ± 0.007	0.04 ± 0.006	0.27 ± 0.05
Site-10	0.013 ± 0.003	0.010 ± 0.001	0.25 ± 0.08	0.08 ± 0.01	10.49 ± 2.15	7.01 ± 0.56	0.02 ± 0.0001	0.13 ± 0.016	0.17 ± 0.03
Site-11	0.013 ± 0.002	0.010 ± 0.001	1.29 ± 0.02	0.22 ± 0.001	13.24 ± 20.26	7.72 ± 0.60	0.06 ± 0.001	0.04 ± 0.011	0.19 ± 0.05
Site-12	0.010 ± 0.003	0.010 ± 0.001	0.10 ± 0.03	0.33 ± 0.001	11.00 ± 1.68	6.75 ± 0.70	0.04 ± 0.004	0.03 ± 0.007	0.18 ± 0.07
Site-13	0.016 ± 0.001	0.003 ± 0.0001	0.07 ± 0.01	0.20 ± 0.003	3.86 ± 0.33	8.59 ± 0.44	0.07 ± 0.007	0.01 ± 0.006	0.26 ± 0.02

Data expressed as mean ± SE (n = 6).

**Table 9:** Concentrations (mg kg^−1^) of V, Cr, Mn, Co, Ni, Cu, Zn, Mo and Ag in kidney tissue of *Labeo rohita* collected from 13 different sites of East Kolkata Wetland.

Sites	V	Cr	Mn	Co	Ni	Cu	Zn	Mo	Ag
Site-1	0.07 ± 0.003	1.35 ± 0.10	0.65 ± 0.09	0.05 ± 0.001	1.21 ± 0.34	3.80 ± 0.24	17.55 ± 1.47	0.09 ± 0.011	0.36 ± 0.05
Site-2	0.15 ± 0.02	0.90 ± 0.15	0.62 ± 0.05	0.03 ± 0.001	1.00 ± 0.29	7.07 ± 0.52	16.81 ± 2.13	0.20 ± 0.08	0.22 ± 0.04
Site-3	0.08 ± 0.004	1.00 ± 0.09	0.52 ± 0.14	0.05 ± 0.001	0.62 ± 0.19	3.68 ± 0.64	9.35 ± 0.65	0.09 ± 0.007	0.02 ± 0.001
Site-4	0.07 ± 0.006	1.16 ± 0.38	0.49 ± 0.09	0.03 ± 0.001	1.08 ± 0.31	3.51 ± 0.10	15.42 ± 0.51	0.10 ± 0.07	0.10 ± 0.05
Site-5	0.05 ± 0.01	0.41 ± 0.08	0.73 ± 0.08	0.02 ± 0.001	0.45 ± 0.32	3.85 ± 0.75	21.87 ± 1.29	0.06 ± 0.003	0.02 ± 0.001
Site-6	0.05 ± 0.01	0.43 ± 0.07	0.41 ± 0.06	0.06 ± 0.02	0.50 ± 0.08	3.57 ± 0.24	14.30 ± 1.46	0.05 ± 0.005	0.16 ± 0.04
Site-7	0.07 ± 0.01	0.47 ± 0.12	0.62 ± 0.07	0.02 ± 0.001	0.69 ± 0.05	4.37 ± 0.44	21.04 ± 2.31	0.12 ± 0.009	0.11 ± 0.06
Site-8	0.12 ± 0.001	0.55 ± 0.16	0.89 ± 0.08	0.09 ± 0.01	0.60 ± 0.27	3.62 ± 0.15	15.27 ± 0.92	0.09 ± 0.06	0.99 ± 0.32
Site-9	0.09 ± 0.06	0.64 ± 0.09	0.48 ± 0.03	0.03 ± 0.01	0.69 ± 0.34	9.99 ± 0.80	15.07 ± 0.91	0.07 ± 0.01	1.15 ± 0.17
Site-10	0.10 ± 0.01	0.69 ± 0.06	0.78 ± 0.04	0.16 ± 0.06	0.60 ± 0.20	3.71 ± 0.41	20.95 ± 1.65	0.06 ± 0.02	0.02 ± 0.01
Site-11	0.05 ± 0.03	0.55 ± 0.05	0.34 ± 0.08	0.05 ± 0.02	0.52 ± 0.16	3.38 ± 0.25	12.40 ± 0.88	0.05 ± 0.08	0.02 ± 0.001
Site-12	0.07 ± 0.01	0.49 ± 0.05	0.64 ± 0.20	0.11 ± 0.04	0.62 ± 0.20	3.52 ± 0.35	24.14 ± 1.60	0.07 ± 0.06	0.02 ± 0.01
Site-13	0.06 ± 0.001	0.47 ± 0.16	0.51 ± 0.03	0.05 ± 0.01	1.05 ± 0.31	3.48 ± 0.51	21.52 ± 1.55	0.07 ± 0.07	0.05 ± 0.05

Data expressed as mean ± SE (n = 6).

**Table 10:** Concentrations (mg kg^−1^) of Ga, Ge, Se, Sr, Sn, Cd, Hg and Pb in *kidney* tissue of *Labeo rohita* collected from 13 different sites of East Kolkata Wetland.

Sites	Ga	Ge	As	Se	Sr	Sn	Cd	Hg	Pb
Site-1	0.0047 ± 0.0010	0.020 ± 0.03	0.17 ± 0.01	1.27 ± 0.77	0.25 ± 0.02	10.92 ± 1.29	0.16 ± 0.003	0.041 ± 0.010	0.22 ± 0.01
Site-2	0.0074 ± 0.005	0.060 ± 0.02	0.15 ± 0.07	0.37 ± 0.11	0.41 ± 0.04	5.57 ± 0.53	0.09 ± 0.004	0.026 ± 0.016	0.54 ± 0.05
Site-3	0.0047 ± 0.017	0.010 ± 0.01	0.09 ± 0.001	0.12 ± 0.14	0.25 ± 0.03	8.36 ± 1.18	0.05 ± 0.03	0.022 ± 0.011	0.24 ± 0.02
Site-4	0.0080 ± 0.031	0.007 ± 0.02	0.08 ± 0.01	0.12 ± 0.017	0.31 ± 0.03	8.44 ± 1.42	0.05 ± 0.003	0.015 ± 0.004	0.26 ± 0.01
Site-5	0.0102 ± 0.010	0.016 ± 0.03	0.10 ± 0.09	0.10 ± 0.058	0.32 ± 0.05	8.60 ± 1.18	0.04 ± 0.01	0.015 ± 0.004	0.14 ± 0.04
Site-6	0.0030 ± 0.003	0.010 ± 0.04	0.11 ± 0.01	1.12 ± 0.10	0.21 ± 0.01	7.87 ± 1.15	0.07 ± 0.01	0.019 ± 0.007	0.18 ± 0.06
Site-7	0.0072 ± 0.017	0.012 ± 0.05	0.09 ± 0.001	0.18 ± 0.01	0.26 ± 0.02	8.47 ± 0.56	0.21 ± 0.04	0.019 ± 0.013	0.16 ± 0.02
Site-8	0.0094 ± 0.011	0.011 ± 0.04	0.07 ± 0.002	0.35 ± 0.029	1.00 ± 0.02	7.88 ± 1.20	0.04 ± 0.07	0.015 ± 0.007	0.36 ± 0.06
Site-9	0.0110 ± 0.019	0.018 ± 0.04	0.12 ± 0.001	0.32 ± 0.02	0.49 ± 0.01	8.05 ± 0.43	0.04 ± 0.02	0.033 ± 0.011	0.18 ± 0.04
Site-10	0.0044 ± 0.007	0.009 ± 0.03	0.15 ± 0.001	0.49 ± 0.04	0.29 ± 0.03	5.87 ± 0.81	5.46 ± 0.38	0.033 ± 0.011	0.16 ± 0.02
Site-11	0.0028 ± 0.010	0.009 ± 0.02	0.14 ± 0.09	0.42 ± 0.02	0.17 ± 0.02	8.58 ± 0.48	0.04 ± 0.08	0.037 ± 0.010	0.20 ± 0.04
Site-12	0.0055 ± 0.012	0.017 ± 0.02	0.14 ± 0.001	0.33 ± 0.07	0.24 ± 0.02	7.03 ± 0.76	0.72 ± 0.21	0.022 ± 0.013	0.28 ± 0.07
Site-13	0.0047 ± 0.015	0.013 ± 0.05	0.13 ± 0.002	0.12 ± 0.003	0.22 ± 0.04	8.39 ± 0.53	0.05 ± 0.02	0.022 ± 0.06	0.45 ± 0.06

Data expressed as mean ± SE (n = 6).

**Table 11:** Concentrations (mg kg^−1^) of V, Cr, Mn, Co, Ni, Cu, Zn, Mo and Ag in liver tissue of *Labeo rohita* collected from 13 different sites of East Kolkata Wetland.

Sites	V	Cr	Mn	Co	Ni	Cu	Zn	Mo	Ag
Site-1	0.15 ± 0.02	0.77 ± 0.12	0.43 ± 0.04	0.02 ± 0.01	0.61 ± 0.29	6.41 ± 0.86	18.73 ± 1.61	0.20 ± 0.15	0.40 ± 0.20
Site-2	0.13 ± 0.02	0.53 ± 0.07	2.56 ± 0.25	0.07 ± 0.06	0.75 ± 0.03	4.61 ± 0.46	13.97 ± 1.42	0.11 ± 0.07	0.05 ± 0.05
Site-3	0.07 ± 0.01	0.45 ± 0.12	0.53 ± 0.02	0.03 ± 0.02	0.91 ± 0.18	7.01 ± 0.80	17.39 ± 2.28	0.13 ± 0.06	0.19 ± 0.09
Site-4	0.09 ± 0.03	0.49 ± 0.05	0.73 ± 0.13	0.03 ± 0.02	0.72 ± 0.11	3.40 ± 0.72	14.27 ± 3.56	0.05 ± 0.05	0.04 ± 0.04
Site-5	0.08 ± 0.02	0.36 ± 0.05	1.06 ± 0.20	0.03 ± 0.01	1.31 ± 0.18	3.42 ± 0.59	13.49 ± 2.71	0.09 ± 0.06	0.11 ± 0.11
Site-6	0.05 ± 0.03	0.69 ± 0.13	1.20 ± 0.10	0.04 ± 0.02	1.41 ± 0.13	4.22 ± 0.34	25.05 ± 4.68	0.25 ± 0.11	0.08 ± 0.06
Site-7	0.08 ± 0.02	0.51 ± 0.13	0.47 ± 0.07	0.03 ± 0.02	0.83 ± 0.06	3.44 ± 0.73	22.22 ± 3.67	0.07 ± 0.00	0.04 ± 0.07
Site-8	0.06 ± 0.02	0.58 ± 0.16	0.94 ± 0.13	0.03 ± 0.02	0.64 ± 0.31	4.04 ± 0.36	23.27 ± 5.12	0.17 ± 0.21	0.04 ± 0.06
Site-9	0.12 ± 0.03	0.48 ± 0.05	0.56 ± 0.15	0.02 ± 0.02	0.75 ± 0.06	13.57 ± 2.58	19.39 ± 3.29	0.12 ± 0.05	0.54 ± 0.18
Site-10	0.07 ± 0.03	0.50 ± 0.02	0.71 ± 0.21	0.03 ± 0.02	0.76 ± 0.02	6.56 ± 0.58	21.58 ± 2.89	0.19 ± 0.03	0.17 ± 0.13
Site-11	0.10 ± 0.04	0.52 ± 0.12	1.04 ± 0.18	0.03 ± 0.02	0.66 ± 0.11	12.83 ± 0.79	22.84 ± 1.86	0.19 ± 0.06	0.71 ± 0.25
Site-12	0.07 ± 0.01	0.44 ± 0.10	0.44 ± 0.09	0.05 ± 0.01	0.45 ± 0.38	3.33 ± 0.30	20.91 ± 2.49	0.12 ± 0.09	0.03 ± 0.02
Site-13	0.07 ± 0.06	0.49 ± 0.11	1.43 ± 0.18	0.04 ± 0.02	0.90 ± 0.46	3.36 ± 0.05	26.83 ± 3.68	0.09 ± 0.09	0.03 ± 0.02

Data expressed as mean ± SE (n = 6).

**Table 12:** Concentrations (mg kg^−1^) of Ga, Ge, Se, Sr, Sn, Cd, Hg and Pb in liver tissue of *Labeo rohita* collected from 13 different sites of East Kolkata Wetland.

Sites	Ga	Ge	As	Se	Sr	Sn	Cd	Hg	Pb
Site-1	0.009 ± 0.03	0.06 ± 0.02	0.19 ± 0.07	0.18 ± 0.044	0.17 ± 0.05	6.15 ± 0.74	0.05 ± 0.01	0.03 ± 0.07	0.34 ± 0.03
Site-2	0.019 ± 0.06	0.20 ± 0.14	0.17 ± 0.11	5.29 ± 1.85	0.40 ± 0.02	4.68 ± 0.35	0.13 ± 0.08	0.02 ± 0.17	0.34 ± 0.04
Site-3	0.006 ± 0.01	0.02 ± 0.03	0.12 ± 0.05	0.65 ± 0.26	0.25 ± 0.02	5.45 ± 0.76	0.08 ± 0.08	0.03 ± 0.16	0.33 ± 0.05
Site-4	0.007 ± 0.01	0.02 ± 0.04	0.19 ± 0.13	0.10 ± 0.50	0.26 ± 0.02	7.81 ± 1.09	0.08 ± 0.05	0.01 ± 0.06	0.33 ± 0.03
Site-5	0.003 ± 0.00	0.03 ± 0.03	0.13 ± 0.09	0.28 ± 0.44	0.31 ± 0.04	0.10 ± 0.08	0.07 ± 0.05	0.02 ± 0.06	0.32 ± 0.07
Site-6	0.007 ± 0.01	0.02 ± 0.05	0.14 ± 0.05	0.95 ± 0.50	0.25 ± 0.06	1.68 ± 0.55	0.12 ± 0.13	0.01 ± 0.06	0.31 ± 0.06
Site-7	0.007 ± 0.00	0.04 ± 0.04	0.16 ± 0.18	0.70 ± 0.30	0.29 ± 0.05	5.95 ± 0.61	0.07 ± 0.05	0.02 ± 0.13	0.33 ± 0.04
Site-8	0.007 ± 0.01	0.13 ± 0.07	0.18 ± 0.09	48.54 ± 9.83	0.28 ± 0.03	1.10 ± 0.07	0.10 ± 0.07	0.01 ± 0.06	0.17 ± 0.03
Site-9	0.006 ± 0.00	0.04 ± 0.06	0.16 ± 0.08	1.07 ± 0.93	0.35 ± 0.04	3.88 ± 0.68	0.08 ± 0.04	0.02 ± 0.19	0.17 ± 0.03
Site-10	0.008 ± 0.01	0.02 ± 0.05	0.19 ± 0.11	0.87 ± 2.10	0.30 ± 0.02	2.38 ± 0.48	0.07 ± 0.03	0.03 ± 0.10	0.14 ± 0.03
Site-11	0.010 ± 0.02	0.11 ± 0.08	0.12 ± 0.19	0.79 ± 1.46	0.39 ± 0.08	0.36 ± 0.07	0.08 ± 0.04	0.06 ± 0.13	0.18 ± 0.01
Site-12	0.006 ± 0.01	0.02 ± 0.02	0.13 ± 0.08	0.47 ± 0.31	0.26 ± 0.10	7.51 ± 0.59	0.06 ± 0.05	0.13 ± 0.06	0.16 ± 0.05
Site-13	0.006 ± 0.02	0.02 ± 0.03	0.20 ± 0.07	0.67 ± 0.73	0.30 ± 0.05	6.69 ± 0.43	0.13 ± 0.05	0.06 ± 0.21	0.17 ± 0.04

Data expressed as mean ± SE (n = 6).

**Table 13:** Concentrations (mg kg^−1^) of V, Cr, Mn, Co, Ni, Cu, Zn, Mo and Ag in brain tissue of *Labeo rohita* collected from 13 different sites of East Kolkata Wetland.

Sites	V	Cr	Mn	Co	Ni	Cu	Zn	Mo	Ag
Site-1	0.09 ± 0.04	0.45 ± 0.13	0.48 ± 0.11	0.03 ± 0.03	0.67 ± 0.19	3.23 ± 0.30	11.11 ± 0.36	0.06 ± 0.012	0.03 ± 0.04
Site-2	0.05 ± 0.02	0.40 ± 0.04	0.33 ± 0.03	0.02 ± 0.02	0.97 ± 0.06	3.31 ± 0.44	12.24 ± 0.61	0.04 ± 0.04	0.02 ± 0.01
Site-3	0.13 ± 0.06	0.55 ± 0.23	0.96 ± 0.12	0.04 ± 0.01	0.56 ± 0.25	3.00 ± 0.09	10.92 ± 1.33	0.04 ± 0.01	0.02 ± 0.02
Site-4	0.06 ± 0.04	0.40 ± 0.10	0.37 ± 0.17	0.01 ± 0.00	0.70 ± 0.18	3.24 ± 0.51	10.00 ± 0.52	0.05 ± 0.04	0.01 ± 0.01
Site-5	0.06 ± 0.05	0.39 ± 0.02	0.29 ± 0.13	0.01 ± 0.01	0.61 ± 0.12	2.90 ± 0.86	9.20 ± 2.51	0.03 ± 0.01	0.01 ± 0.01
Site-6	0.07 ± 0.02	0.44 ± 0.12	0.52 ± 0.04	0.02 ± 0.03	0.71 ± 0.29	3.28 ± 0.52	14.92 ± 2.47	0.05 ± 0.01	0.04 ± 0.02
Site-7	0.08 ± 0.05	0.59 ± 0.10	0.44 ± 0.01	0.02 ± 0.04	0.54 ± 0.17	2.96 ± 0.23	11.88 ± 2.27	0.09 ± 0.08	0.02 ± 0.03
Site-8	0.06 ± 0.01	0.42 ± 0.12	0.38 ± 0.05	0.02 ± 0.01	0.69 ± 0.17	3.36 ± 0.58	11.60 ± 2.52	0.06 ± 0.00	0.05 ± 0.01
Site-9	0.06 ± 0.03	0.40 ± 0.05	0.43 ± 0.12	0.02 ± 0.01	0.56 ± 0.03	3.48 ± 0.27	10.60 ± 2.03	0.07 ± 0.01	0.01 ± 0.02
Site-10	0.03 ± 0.04	0.35 ± 0.06	0.24 ± 0.01	0.03 ± 0.04	0.50 ± 0.18	3.11 ± 0.60	8.29 ± 0.49	0.04 ± 0.07	0.01 ± 0.00
Site-11	0.04 ± 0.04	0.33 ± 0.13	0.30 ± 0.11	0.02 ± 0.03	0.52 ± 0.19	3.32 ± 0.30	8.53 ± 0.36	0.11 ± 0.01	0.020 ± 0.04
Site-12	0.06 ± 0.02	0.50 ± 0.04	0.37 ± 0.03	0.02 ± 0.02	0.64 ± 0.06	3.30 ± 0.44	8.20 ± 0.61	0.08 ± 0.04	0.014 ± 0.01
Site-13	0.05 ± 0.06	0.38 ± 0.23	0.38 ± 0.12	0.02 ± 0.01	0.51 ± 0.25	3.24 ± 0.09	16.06 ± 1.33	0.05 ± 0.06	0.007 ± 0.001

Data expressed as mean ± SE (n = 6).

**Table 14:** Concentrations (mg kg^−1^) of Ga, Ge, Se, Sr, Sn, Cd, Hg and Pb in brain tissue of *Labeo rohita* collected from 13 different sites of East Kolkata Wetland.

Sites	Ga	Ge	As	Se	Sr	Sn	Cd	Hg	Pb
Site-1	0.006 ± 0.003	0.02 ± 0.07	0.14 ± 0.11	0.17 ± 0.44	0.22 ± 0.08	8.08 ± 1.35	0.04 ± 0.01	0.03 ± 0.01	0.31 ± 0.06
Site-2	0.003 ± 0.005	0.02 ± 0.05	0.15 ± 0.17	0.20 ± 0.29	0.19 ± 0.02	8.07 ± 0.47	0.03 ± 0.001	0.04 ± 0.01	0.11 ± 0.04
Site-3	0.007 ± 0.006	0.02 ± 0.01	0.15 ± 0.12	0.74 ± 0.44	2.82 ± 0.11	7.31 ± 0.80	0.02 ± 0.001	0.04 ± 0.20	0.17 ± 0.06
Site-4	0.004 ± 0.011	0.02 ± 0.02	0.11 ± 0.02	0.64 ± 0.89	0.18 ± 0.06	8.57 ± 0.54	0.10 ± 0.011	0.04 ± 0.01	0.12 ± 0.01
Site-5	0.002 ± 0.000	0.02 ± 0.02	0.11 ± 0.02	1.96 ± 0.77	0.40 ± 0.06	7.68 ± 2.80	0.001 ± 0.001	0.03 ± 0.001	0.09 ± 0.05
Site-6	0.004 ± 0.003	0.01 ± 0.04	0.10 ± 0.11	0.23 ± 0.84	0.67 ± 0.07	8.84 ± 1.07	0.02 ± 0.001	0.04 ± 0.01	0.22 ± 0.05
Site-7	0.007 ± 0.017	0.01 ± 0.01	0.12 ± 0.07	0.15 ± 0.50	0.28 ± 0.03	7.08 ± 1.20	0.02 ± 0.001	0.01 ± 0.002	0.17 ± 0.02
Site-8	0.009 ± 0.012	0.02 ± 0.02	0.11 ± 0.08	0.12 ± 0.17	0.31 ± 0.06	6.47 ± 1.06	0.02 ± 0.006	0.03 ± 0.013	0.12 ± 0.04
Site-9	0.007 ± 0.015	0.01 ± 0.02	0.06 ± 0.02	0.10 ± 0.50	0.27 ± 0.04	7.86 ± 0.87	0.01 ± 0.002	0.01 ± 0.007	0.22 ± 0.03
Site-10	0.001 ± 0.003	0.01 ± 0.02	0.06 ± 0.07	0.22 ± 0.17	2.02 ± 0.13	8.26 ± 0.77	0.01 ± 0.003	0.03 ± 0.004	0.13 ± 0.03
Site-11	0.019 ± 0.003	0.014 ± 0.07	0.10 ± 0.11	0.03 ± 0.44	0.17 ± 0.08	7.58 ± 1.35	0.014 ± 0.009	0.23 ± 0.10	0.14 ± 0.06
Site-12	0.005 ± 0.005	0.009 ± 0.05	0.08 ± 0.17	0.05 ± 0.29	0.22 ± 0.02	5.44 ± 0.47	0.011 ± 0.004	0.11 ± 0.04	0.17 ± 0.04
Site-13	0.004 ± 0.006	0.012 ± 0.01	0.07 ± 0.12	0.12 ± 0.44	0.39 ± 0.11	5.99 ± 0.80	0.036 ± 0.008	0.11 ± 0.020	0.24 ± 0.06

Data expressed as mean ± SE (n = 6).

### Principle component analysis (PCA) and heat map of metal status in fish tissues

**Figure 2:**
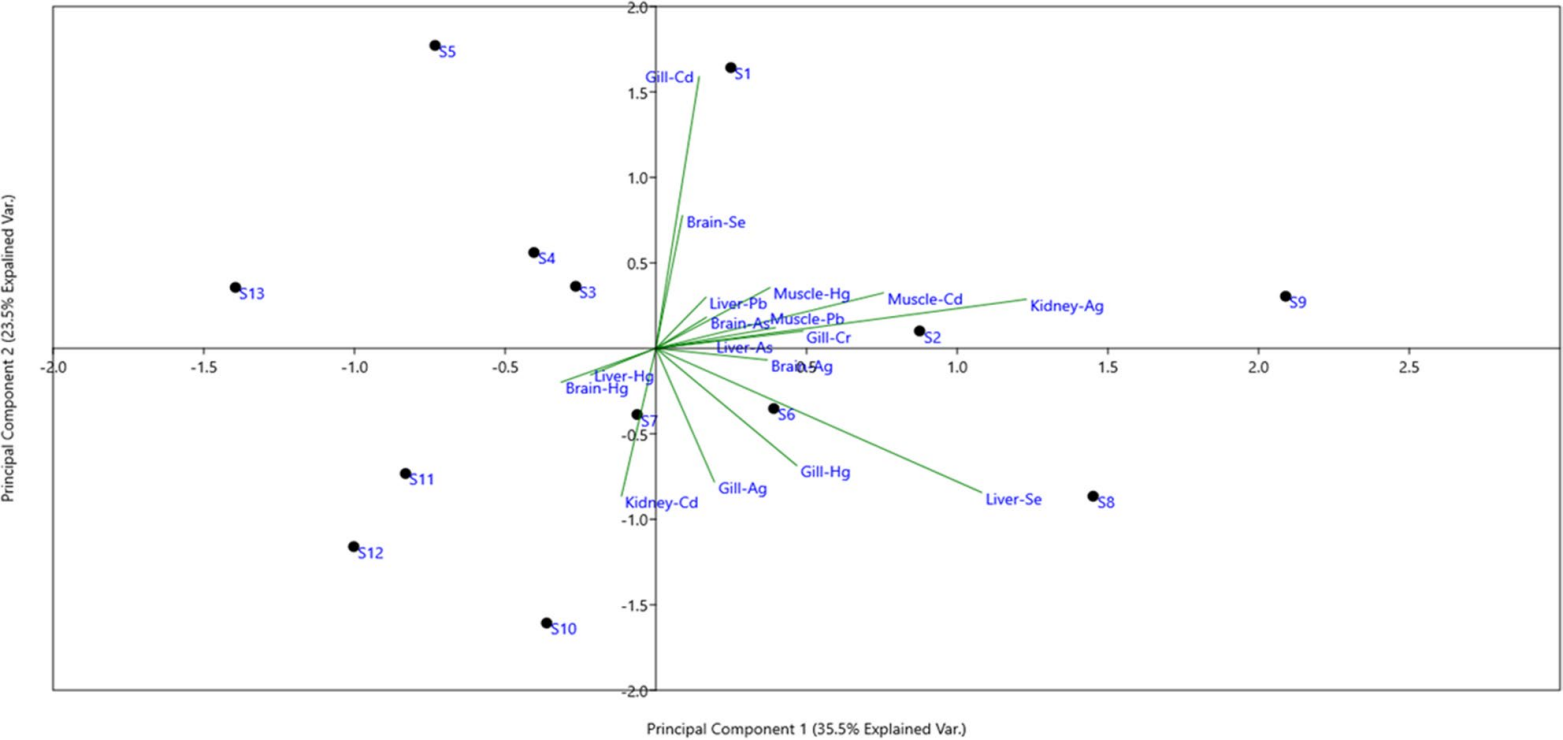
Principal component analysis of metals status in fish tissues, water and soil sediments of East Kolkata Wetland, Ramsar Site.

**Figure 3:**
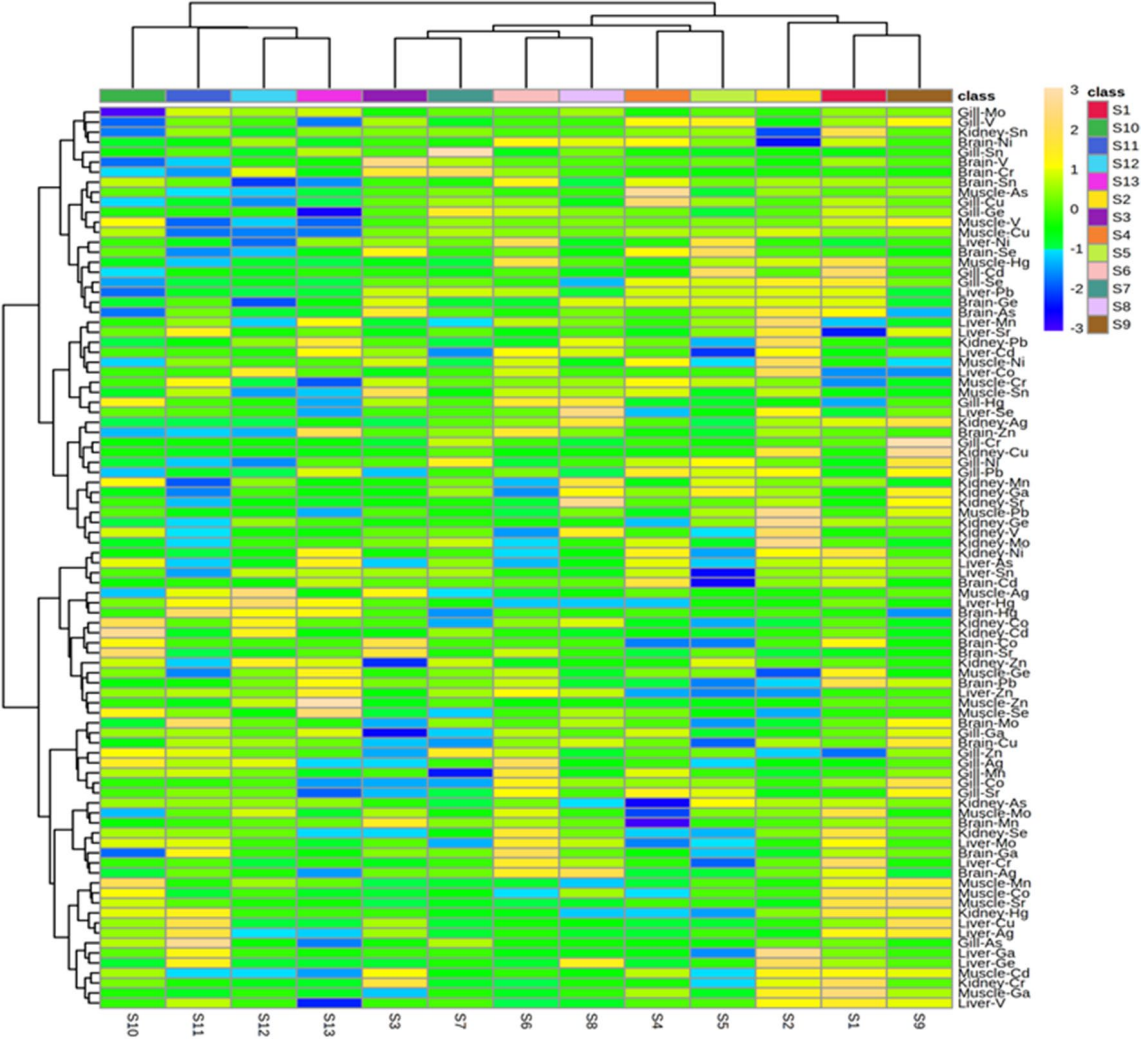
Heat map of metals status of East Kolkata Wetland, Ramsar Site.

The Principal Component Analysis (PCA)-Biplot (Fig. 2) was generated based on the concentration of the metals observed in five fish tissues of 13 sampling sites explaining a total variance of 35.5% across PC1 (Principal Component 1) and 23.5% variance across PC2 (Principal component 2). The biplot vectors of kidney-Ag, muscle-Cd, liver-Se, and gill Hg were observed to be the most important, leading to the segregation of samples in different clusters across PC1 and PC2. As shown in PCA-Biplot, a positive correlation between liver-Hg and brain was observed, whereas a negative correlation was noted between liver Hg and muscle Hg. The clustered heatmap (Fig. 3) on both side clustering (samples-wise and metal concentration) was generated based on the metal concentration observed in different samples of fish tissues. The 13 sampling sites clustered in two major groups based on the elemental concentrations observed in the five fish tissues, however despite the higher concentration of certain elements (higher muscle-As in S4, higher brain-se in S5, higher kidney Ag in S9, higher muscle-Pb in S2) in some of the fish tissues from specific sites, no clustering (either based on the element or the specific tissue type) was noted in the heatmap.

### Environmental assessment (degree of pollution) impact

#### Geoaccumulation (Igeo) index in soil sediments

The Geoaccumulation (Igeo) Index in soil sediments was determined and presented in Table 15. The results showed that East Kolkata Wetland was moderately polluted to strongly polluted with arsenic (As), selenium (Se), and mercury (Hg) with Igeo values for As 2.21, Se 1.31, and Hg 2.70, respectively. Based on soil sediments (Igeo) the East Kolkata Wetland is contaminated.

**Table 15:** Geoaccumulation Index (Igeo) in soil sediments sample collected from 13 different sites of East Kolkata Wetland.

Sites	V	Cr	Mn	Co	Ni	Cu	Zn	As	Se	Sr	Cd	Hg	Pb	Sn
Site-1	− 2.62	− 3.05	− 4.48	− 2.81	− 3.59	− 3.75	− 2.93	1.89	2.76	− 3.36	− 1.36	0.42	− 3.20	− 2.09
Site-2	− 2.78	− 2.77	− 4.81	− 2.96	− 3.54	− 3.47	− 2.92	1.71	1.13	− 3.53	− 2.13	0.42	− 2.96	− 2.27
Site-3	− 2.83	− 2.96	− 4.89	− 2.99	− 3.63	− 2.91	− 1.87	1.63	1.45	− 3.19	− 1.26	1.12	− 1.35	− 1.76
Site-4	− 2.67	− 3.01	− 4.71	− 2.92	− 3.57	− 3.66	− 2.99	1.63	1.14	− 3.61	− 4.17	0.22	− 2.75	− 2.16
Site-5	− 3.00	− 3.43	− 5.29	− 3.65	− 3.86	− 4.05	− 3.41	1.61	0.49	− 4.35	− 4.03	− 1.26	− 3.44	− 1.76
Site-6	− 3.14	− 3.35	− 5.16	− 3.21	− 3.98	− 4.04	− 3.36	1.63	0.79	− 3.33	− 3.17	− 0.42	− 3.27	− 2.25
Site-7	− 3.00	− 3.15	− 4.98	− 3.09	− 3.80	− 3.75	− 3.20	1.42	1.00	− 3.31	− 2.68	− 0.26	− 3.18	− 1.05
Site-8	− 3.86	− 3.79	− 5.17	− 3.43	− 4.18	− 4.27	− 3.52	1.97	1.74	− 3.75	− 3.10	0.12	− 2.92	− 2.37
Site-9	− 3.32	− 3.39	− 4.93	− 3.31	− 3.77	− 3.34	− 3.03	2.04	1.95	− 3.97	− 2.36	− 1.26	− 2.49	− 1.90
Site-10	− 2.99	− 3.40	− 4.70	− 3.20	− 3.92	− 3.56	− 3.10	1.97	2.01	− 3.82	− 1.28	1.17	− 2.38	− 2.21
Site-11	− 3.33	− 2.95	− 4.72	− 3.05	− 3.90	− 3.47	− 2.54	2.06	1.90	− 3.57	− 2.54	1.58	− 3.95	− 2.12
Site-12	− 2.84	− 4.55	− 5.44	− 2.93	− 3.91	− 4.19	− 2.61	1.57	1.00	− 3.53	− 1.79	1.91	− 2.46	− 2.33
Site-13	− 3.10	− 3.21	− 4.70	− 3.67	− 3.88	− 3.74	− 2.05	2.21	1.31	− 3.22	− 1.28	2.70	− 2.00	− 2.82

Data expressed as mean ± SE (n = 6).

#### Contamination index (CI)

The contamination index (CI) of East Kolkata Wetland in water was determined and presented in Table 16. The CI of water for the metals were detected below 0.6, however, the results indicated that water was not contaminated with metals.

**Table 16:** Contamination index (CI) of water in of Cr, Mn, Co, Ni, Cu, Zn, As, Se, Cd, Hg, and Pb in soil sediments sample collected from 13 different sites of East Kolkata Wetland.

Site	Cr	Mn	Co	Ni	Cu	Zn	As	Se	Cd	Hg	Pb	CI
Site-1	0.00189	0.01625	0.00038	0.00428	0.00342	0.00526	0.00456	0.00204	0.00012	0.00469	0.00019	0.52
Site-2	0.00157	0.0015	0.0004	0.00333	0.0032	0.00544	0.00681	0.00639	0.00004	0.00079	0.0002	0.22
Site-3	0.00188	0.00322	0.0003	0.00463	0.00505	0.05456	0.00396	0.0069	0.00986	0.00151	0.00083	0.57
Site-4	0.00132	0.00575	0.00036	0.0035	0.00446	0.03086	0.00671	0.00882	0.0004	0.00031	0.00213	0.22
Site-5	0.00117	0.00389	0.00032	0.00434	0.00451	0.01433	0.01567	0.00537	0.00018	0.00027	0.00061	0.25
Site-6	0.00154	0.00064	0.00035	0.00408	0.00303	0.00497	0.0061	0.00396	0.00007	0.00034	0.00014	0.15
Site-7	0.00138	0.00058	0.00033	0.00291	0.00253	0.0042	0.00419	0.00537	0.00055	0.00116	0.00006	0.23
Site-8	0.00159	0.0012	0.00037	0.00405	0.0023	0.00333	0.00322	0.00831	0.00055	0.00031	0.00009	0.17
Site-9	0.00127	0.00165	0.00041	0.00248	0.00342	0.00289	0.00256	0.00468	0.00027	0.00038	0.00014	0.13
Site-10	0.00165	0.00148	0.00061	0.00164	0.00278	0.00465	0.00315	0.00578	0.00034	0.00075	0.00017	0.18
Site-11	0.00135	0.00162	0.00027	0.00345	0.00212	0.01132	0.00462	0.00468	0.00031	0.00091	0.00023	0.20
Site-12	0.00178	0.00148	0.00036	0.00178	0.00341	0.00256	0.00243	0.00645	0.00046	0.00082	0.00012	0.18
Site-13	0.00162	0.00192	0.00068	0.00249	0.00247	0.00465	0.00275	0.00241	0.00078	0.00076	0.00024	0.16

Data expressed as mean ± SE (n = 6).

### Risk assessments

#### Target hazard quotient (THQ) and cancer risk (CR)

Risk assessment was determined in East Kolkata Wetland using total hazard quotient (THQ) and cancer risk (CR). The results are presented in Table 17. The THQ was determined for metals viz. Cr, Mn, Co, Ni, Cu, Zn, As, Se, Cd, Pb, and Hg in EKW. The value of THQ followed as 0.266, 0.0017, 0.00014, 0.026, 0.072, 0.017, 0.525, 0.876, 3.38, 0.014 and 0.031 respectively. The THQ value results indicated that the concentration of all the metals was well within the acceptable level of risk except for Cd. The cancer risk was also determined for arsenic in this study. The cancer risk for As was obtained as 0.00024. The results of the cancer risk factor indicated that it is well within the acceptable risk and safe for consumption of the fish of EKW.

**Table 17:** Estimated daily intake (EDI), reference dose (RfD), Target Hazard Quotient (THQ), slope factor and cancer risk of Cr, Mn, Co, Ni, Cu, Zn, As, Se, Cd, Pb and Hg from fish muscle sample collected from 13 different sites of East Kolkata Wetland.

	Cr	Mn	Co	Ni	Cu	Zn	As	Se	Cd	Pb	Hg
EDI	0.0008	0.0002	0.000014	0.00052	0.0029	0.0050	0.00016	0.0044	0.0034	0.000054	0.000186
RfD	0.003	0.140	0.100	0.020	0.040	0.300	0.0003	0.005	0.001	0.004	0.006
THQ	0.266	0.00174	0.00014	0.026	0.072	0.017	0.525	0.876	3.377	0.014	0.031
Slope factor							1.5				
Cancer risk							0.00024				

Data expressed as mean ± SE (n = 6).

## Discussion

The East Kolkata Wetland is an important fisheries resource in India and produces more than 10,915 metric tons of fish annually^42,43^. It is the best example of human pressure and natural condition that compete to each other^34^. The present study has evaluated metal status and environmental impact assessment (degree of pollution) in East Kolkata Wetland. The concentration of different metals in muscle, gill, kidney, liver, and brain tissues of *L. rohita* was found well within the safe level as recommended by National and International agencies.

In the present investigation, Vanadium (V) was determined in different fish tissues such as muscle, gill, liver, kidney, brain, and water samples well within the safe level; however, a higher concentration was observed in the soil sediments. The concentration of V was within the safe limit in all the sampling sites. In aquatic animals, the absorption of metal depends upon the biological, chemical, physical, and ecological conditions of water^44,45^. Moreover, fish is the main food item in the human diet. It is the main route of metals to enter the human body to create toxicity effect on human health and cause anemia, inflammation, swelling around the eyes, inflammation of the lungs, cataracts, cognitive deficits, diarrhea, and a decrease in appetite in consumers^45,46^. Moreover, Vanadium (V) also affects the organism’s genetic and evolutionary balance, which can be exchangeable in the fatty tissue of the fish as VO_2+_ and VO_3+._ Generally, it is not stored in the tissue and exists through digestive systems^47,48^. The higher V concentration blocked the enzyme activities and disturbed the nervous and respiratory systems. The high concentration of V can be also an indicator of oil contamination. The beneficial effect of V is important for the suppression of tumor cell growth and cell proliferation^49^.

In the present study, the Chromium (Cr) was determined in different fish tissues viz. muscle, gill, liver, kidney, and brain, in 13 sampling sites EKW. Chromium (Cr) is an essential trace element for a mammal; it has a role in building synthetic complexes with biological ligands, capable of mimicking the activity of glucose tolerance factor^50,51^. However, Cr supplementation helps in therapy to improve insulin sensitivity in diabetic patients^52^ because of its role in glucose metabolism and is also used for fat loss and muscle building^53,54^. The toxic nature of chromium can destabilize the aquatic ecosystem, impacting biota and bioaccumulation. It is also received from natural water from anthropogenic sources such as industrial effluents, sewage, and fertilizers. It is also one of the leading causes of the release of Cr in the environment^55^. It is extensively used in metallurgy, electroplating, paints, pigments, tanning, wood preservation, chemical production, and pulp and paper production^56^. In aquatic organisms, the Cr passes through the gill, accumulating in the brain, liver, kidney, intestine, opercular bone, spleen, and stomach^57,58^. In the present study, the bioaccumulation of Cr in muscle, liver, gill, kidney, and brain was below 1.8 mg kg^−1^ except in the fish muscle site 11. USFDA^59^ states that the MRL level for Cr should be not more than 12–13 mg kg^−1^, and the requirement for humans is 1 µg day^−1^. However, Cr elements are necessary for metabolic purposes in limited quantities.

Manganese (Mn) was highest recorded in gill > liver > kidney > muscle > brain in a sample collected from 13 different sites of East Kolkata Wetland. The gill, liver, kidney, and brain are usually discarded before fish consumption; however, only muscle tissue is consuming part. In the present study, the concentration of Mn was found within the safe limit recommended by different agencies. Mn is naturally present in the environment with low attention, which has played a significant role in the biological system to maintaining metabolic regulation and certain homeostatic^60,61^. It is also an essential co-factor for pyruvate carboxylase and superoxide dismutase. The Mn deficiencies may cause poor reproductive performance, growth retardation, congenital malformations in offspring, and abnormal function of bone and cartilage^62^.

It is well-known fact that cobalt has low accumulation rate in fish tissues^63,64^. Similar results were also obtained in the present investigation as the lowest bioaccumulation in fish tissues. Cobalt is an essential trace metal for human nutrition and integral to vitamin B_12_. Underwood^65^, average daily intake of cobalt, in all forms, is 0.30 to 1.77 mg day^−1^. Co has also been important for blood pressure regulation^66,67^, proper thyroid function^68^ and an activating agent for various enzymatic systems. Congestive heart failure, polycythemia, and anemia were reported in case of excessive ingestion of cobalt^69,70^. In the present study, the highest accumulation of Co was below 0.1 mg kg^−1^ in all the tissues.

In the present investigation, Nickel (Ni) was determined to be below 2 mg kg^−1^ in all the tissues, which is considered a concentration well within the safe level. Ni is essential for normal growth and reproduction in animals, including fish, and at the same time, it shows carcinogenic effects in higher consumption^71^. Generally, it presents in open aquatic systems through natural or human sources, which are circulated into the system by two processes, chemical and physical, using biological transport mechanisms of living organisms^72^. The tool for the transport model of Ni from albumin to histidine via a ternary complex composed of albumin, nickel, and L-histidine. The low molecular weight L-histidine-Ni complex formed crossed biological membranes^73,74^. Inside the mammalian cell, nickel accumulates in the nucleus and nucleolus, disrupting DNA metabolism and causing cross-links and strand breaks^73,74^. The observed redox properties of the nickel-histidine complex are crucial for maximizing the toxicity and carcinogenicity of nickel^75^. Nickel is essential for the normal growth of many microorganisms, including fish^72^. Generally, fish accumulate Ni in different tissues when exposed to elevated levels in their environment^76^ and should not be more than 70–80 mg kg^−1^^77^.

The bioaccumulation of copper in muscle, gill, liver, kidney, and brain tissues were determined. The highest concentration of Cu was determined in the liver tissue as 13.57 mg kg^−1^ at the site of 9, which is a higher concentration than other tissues. Cu is essential for many enzymatic processes, including the synthesis of hemoglobin. It is a cofactor for several enzymatic systems in most living organisms. The bioaccumulation of Cu has been related to Cu toxicity^78^, and it should not be more than 30 mg kg^−1^^72^. However, a very high intake of Cu will cause adverse health problems^79^. These effects result in biochemical and physiological changes in fish blood and can be an indicator of the physiological state of fish^80^. However, in the present study bioaccumulation of Cu was highest in the liver, followed by kidney, muscle, gill, and brain tissues.

Zinc (Zn) is an essential trace element for maintaining enzymatic and physiological systems in animals, fish, and humans. In this study, the concentration was higher in gill, liver, kidney, and muscle tissues at 29, 26, 24 and 24 mg kg^−1^ in sites 10, 13, 12 and 13, respectively. The concentration of Zn was higher but well within the safe level for consumption. Zn is a major component of nucleic acid synthesis and many enzymatic systems^81^, immune system, neurotransmission and cell signaling^82^. It is also the most common metal pollutant accumulated in the different fish tissues and able to bio-magnify in the food chain system. It has direct toxicity along with other metals in case of increased waterborne levels^83^. Generally, the toxicity starts with gill tissues due to its first exposure to water, whereas Ca^2+^ uptake is disrupted, leading to hypocalcemia and eventual death^8,83,84^. The most susceptible organ for bioaccumulation of Zn is the kidney, which has a tremendous role in animals and human being^85^. The toxicity is also reflected in the fish’s survival, growth, reproduction, and hatching.

Molybdenum (Mo) is the least toxic and essential for the growth and development of animals, including fish. There is scant research work conducted in the area of Mo toxicity in fish. Generally, it is present in the molybdopterin cofactor in the cell and also known function in xanthine oxidase/dehydrogenase, aldehyde oxidase and sulfite oxidase^86^. It is crucial for protection against copper, mercury, and other metals toxicity which also has anti-carcinogenic properties. In this study, the Mo bioaccumulation has been performed in muscle, gill, kidney, liver, and brain, which is present in the very least concentration. A US study recommends 120–240 µg day^−1^ with an average dose of 180 µg day^−1^ for daily intake^87^.

Arsenic toxicity is generally reported in some parts of India and the world. Arsenic contamination spreads through natural processes such as weathering. It was also used to kill aquatic plants that are generally barriers during hook-and-line fishing^88^. In the present study, the bioaccumulation of As in muscle, liver, gill, brain, and kidney were determined well within the safe level from sampling sites 1–13. It showed that the fish from sites 1–13 were not contaminated with arsenic toxicity. Moreover, As is accumulated in the soil sediments in some sampling sites. Arsenic metabolism occurs in the liver and kidney tissues^89^. Arsenic can accumulate in metalloid form and finally reach a toxic level in tissues and causes several diseases^90^. However, arsenic bioaccumulation primarily occurs in the fish's retina, liver, and kidney tissues, which can alter the fish immune system by suppressing several antibodies^91^. Arsenic toxicity is a highly complex process as it occurs in two forms, As (III) and As (V), and both are found in aquatic ecosystems. The As (III) is slowly oxidized to As V under an oxygen-rich situation, and As III is more toxic than As (V)^91^.

The selenium was determined in the different fish tissues, viz. muscle, gill, liver, kidney, and brain, from sampling sites 1–13 East Kolkata Wetland. In muscle tissues, the concentration of selenium was below 1.21 mg kg^−1^, whereas in gill and liver tissues, the highest concentration was obtained at 13.73 and 48.54 mg kg^−1^ at sites 1 and 9, respectively. Selenium has an essential role in normal growth, physiological function, and maintenance of the homeostatic of the animal at lowered concentrations^92^. It is one of the essential elements of the antioxidant defense system, thyroid hormone metabolism, and spermatogenesis^93^ in fish. Se is also important in protecting against the toxicity of exogenous metals such as Hg^41,94^ and reduces the availability of Hg as methylmercury, blocking it in insoluble compounds^94^. It is distributed throughout the environment in ground and surface waters at concentrations between 0.1 and 0.4 μg/L of Se^95^. The liver, gonad, and kidney tissues are prime target for bioaccumulation of Se^88^. However, it becomes more toxic when it crosses the upper threshold concentration^96^. Excess Se, even as low as 3–8 ppb, in the water, can cause numerous life-threatening changes in freshwater fish^88,97^.

Cadmium (Cd) is a naturally occurring non-essential trace element, and its tendency to bioaccumulate in living organisms, often at hazardous levels, raises environmental concern^98^. The Cd production, consumption, and environmental emissions have increased dramatically during the current period, leading to contamination of aquatic habitats^10^. The use of cadmium containing fertilizer, agricultural chemicals, pesticides, and sewage sludge in farmland, might also contribute to water contamination. Cd and its compounds are highly water soluble compared to other metals. However, their bioavailability is very high and tends to bioaccumulate in fish tissue. The interaction properties of Cd with essential nutrients are the leading cause of toxicity. The kidney, liver, gill^99^, and other tissues are the prime organs for bioaccumulation of Cd. Moreover, cadmium inhibits calcium uptake in gills^100^. It may alter the metabolism of essential trace elements by affecting the normal tissue distribution of trace elements such as Zn and Cu^101^.

Mercury (Hg) is the most toxic heavy metal in the world. The fish received Hg mainly through contaminated food, which can be determined by fish size, diet, ecological parameters, and water quality parameters such as a rise in water temperatures attributed to climate change may stimulate the methylation of Hg. The major Hg toxicity occurred due to the organic form of methylmercury (MeHg^+^)^96^ and estimated that 70 to 100% of the Hg in fish is present as MeHg^+^. The methylmercury has created pollution in Minimata Bay, Japan^102^. The liver is a prime organ for binding, storage, and redistribution of Hg in fish tissues^103^. The mercury and its compounds could be retained in the tissues of fish for long periods, resulting in irreversible damages, such as neurological impairment and lesions, behavioral and cognitive changes, ataxia, as well as convulsions, in addition to its harmful effect on reproduction^104^. At very low concentrations, mercury reduces spermatozoa’s viability, reduces egg production, and affects the survival rate of developing eggs and fry^105^.

Lead is a persistent toxic element with no biological role and causes carcinogenic effects in aquatic systems and the human population^106^. In aquatic systems, Pb maybe comes from industrial and smelter discharges, lead-containing pesticides, precipitation, the fallout of lead dust, street runoff, and municipal wastewater. Fish can bioaccumulate Pb from water and diet^106^. However, there is evidence that Pb accumulation in fish most probably originated from contaminated water rather than diet^107^. The trace elements such as gallium (Ga), germanium (Ge), strontium (Sr), and tin (Sn) has also impacted aquatic organism, including fishes.

The concentrations of V, Cr, Mn, Co, Ni, Cu, Zn, Mo, Ag, Ga, Ge, As, Se, Sr, Sn, Cd, Hg, and Pb were detected in the water sample of East Kolkata Wetland. Still, it does not exceed the maximum residual level (MRL), whereas As Cd and Hg were higher at 15.67, 9.86, and 4.69 µg L^−1^ at sampling sites 5, 3, and 1, respectively. Most of the elements throughout the sampling sites have low due to absorption to suspended matter and sediment particles, which rapidly remove the elements from the water column^108^. Similarly, As and Pb were reached up to 7.65 mg kg^−1^, and 10.60 mg kg^−1^ of soil sediment at one 1 sampling site**, **and the rest were well within recommended level. Soil sediments are the major storing site of metals, holding more than 99% of the total amount of metals in the aquatic systems^109^. In addition, heavy rainfall leads to farm draining. It results in large amounts of pesticide-containing metal compounds, which are brought via surface runoff from the farms to the river and increases agriculture pollution. Trace element contaminated sediment may act as a secondary pollution source for the aquatic ecosystem, and elements concentrations in sediment are also helpful for estimating pollution trends^110^.

The geoaccumulation index (Igeo) of East Kolkata Wetland soil sediment was determined as per the Muller^111^ method. The present results showed that V, Cr, Mn, Co, Ni, C, Zn, As, Se, Sr, Cd, Hg, Pb, and Sn in soil sediments were found well within the safe level (Igeo < 0), but in case of Se, As and Hg, Igeo are 1.31, 2.21 and 2.7 respectively. However, the Se Igeo value indicates moderately polluted, while As and Cd Igeo indicates moderately to strongly polluted. It is well understood that the metal accumulation is more in the case of sediment compared to water and fish.

The contamination index (CI) of water was compared with SON^37^ and WHO^38^. The present study of CI in water was well within the safe level as the value of CI is below 0.6. Moreover, this result indicates that water from East Kolkata Wetland is safe for fish culture.

In the present investigation, the target hazard quotients (THQs) of 16 metals were determined in the East Kolkata Wetland using estimated daily intake and reference dose concerning fish consumption. The results showed that no health risk was involved with respect to heavy metal exposure on the EKW. THQs values were obtained below 1, which indicates the safe level of THQ except for cadmium. The major toxic metal such as Pb, Cd, Hg, and As for tolerable intake limit recommended by different agencies viz. FAO/WHO. The doses for Pb intake in adults are 25 µg kg^−1^ body weight and 3.57 µg kg^−1^ per day^35^. Similarly, Cd 57–71 µg day^−1^ through fish consumption has been recommended by FAO^112^. The provisional tolerable daily intake (PTDI) of As, Cr, Mn, Co, Ni, Cu, Zn, Se, and Hg are 2.14, 3, 140, 1.2, 5, 500, 1000, 4, and 0.7 µg kg^−1^ body weight/day respectively has been standardized by the different agency (FAO/WHO). In the present study, the THQ for all the metals well within the 1 except Cd, the value of Cd is 3.37, which mean it is contaminated with Cd. The cancer risk of arsenic in the present study is well within the range (2.4 × 10^−5^), which means the risk is acceptable.

In the present investigation, PCA revealed that the separation was obtained at 35.5% and 23.5 percent in PC 1 and PC 2, respectively. It also observed that Ag in the kidney, Cd in muscle, Se in the liver, and Hg in the gill were the most important, leading to the segregation of samples in different clusters across PC1 and PC2. Similarly, the heat map disclosed both side clustering (samples-wise and metal concentration) on the concentration of the metal in different fish tissues. It was also observed that, despite of the higher concentration of certain elements (higher muscle-As in S4, higher brain-se in S5, higher kidney Ag in S9, higher muscle-Pb in S2) in few of the fish tissues from specific sites, no clustering (either based on the element or the specific tissue type) was noted in the heatmap.

## Conclusion

The East Kolkata Wetland (EKW) is an important ecological site as Ramsar. The present investigation assessed 16 metals in different fish tissues, water samples, and soil sediments at 13 samplings sites in EKW. In this study, environmental assessment impact (degree of pollution) was determined in the form of a geoaccumulation index (Igeo) in soil sediments, and contamination index (CI) in water samples were determined. The fish culture in EKW is provided to West Bengal and other states; however, risk assessment was also determined in the form of Target hazard quotient (THQ) and cancer risk factors. The metals viz. Chromium, Vanadium, Cobalt, Manganese, Copper, Nickel, Zinc, Silver, Molybdenum, Arsenic, Selenium, Tin, Strontium, Cadmium, Mercury, and Lead were determined in muscle, gill, liver, kidney, and brain in 13 sampling sites. The results revealed that bioaccumulation of metals in muscle tissues is well within the safe level as recommended by National and International agencies. Considering that muscle tissue is essential for metals assessment because this is the edible part, and if contaminated, it will cause an adverse effect on consumers. Indeed, the metal contamination in other tissues such as the liver, gill, kidney, and brain were also found below the toxic level. In the case of metals, concentration in water samples was also found below the toxic level. The Igeo index of V, Cr, Mn, Co, Ni, C, Zn, As, Se, Sr, Cd, Hg, Pb and Sn in soil sediments were found under well within the safe level (Igeo < 0), but in case Se, As and Hg, Igeo are 1.31, 2.21 and 2.7 respectively. However, the Se Igeo value indicates moderately polluted, while As and Cd Igeo indicates moderately to strongly polluted. It is well understood that the metal accumulation is more in the case of sediment compared to water and fish. Based on the results of CI in water, the EKW is safe for culture. The risk assessment in the form of THQ and cancer risk factor results revealed that THQ for all the metals was found below 1, indicating the safe level of THQ except cadmium. Similarly, the cancer risk factor was also determined for arsenic which was also found well within the safe level. The present investigation is the first study which deals with assessment of 18 metals and health hazards in human being including risk assessment with respect to cancer risk factor, THQ, slope factor, and RFD in Wetlands in India. Hence, it is recommended that fish from East Kolkata Wetland is safe for consumption.

## Supplementary Information

Supplementary Table S1.

## Data Availability

The datasets used and analyzed during the current study are available from the corresponding author upon reasonable request.
