# Supplementary material for: Health risk assessment and metal contamination in fish, water and soil sediments in the East Kolkata Wetlands, India, Ramsar site
Source: Sci Rep. 2023 Jan 27;13:1546. doi: 10.1038/s41598-023-28801-y (PMC9883242; doi:10.1038/s41598-023-28801-y)
Supplement: Supplementary file 1 — Supplementary Table S1. [file 41598_2023_28801_MOESM1_ESM.doc]

Supplementary Table 1: Global position system of sampling sites of Eastern Kolkata Wetland

| Site No. | Latitude | Longitude |
| --- | --- | --- |
| 1 | N22.33.172 | E88.26.600 |
| 2 | N22.32.308 | E88.25.973 |
| 3 | N22.34.105 | E88.25.760 |
| 4 | N22.33.411 | E88.26.373 |
| 5 | N22.32.880 | E88.27.112 |
| 6 | N22.32.494 | E88.27.421 |
| 7 | N22.32.783 | E88.27.638 |
| 8 | N22.33.785 | E88.25.124 |
| 9 | N22.32.142 | E88.27.509 |
| 10 | N 22.31.745 | E 88.28.014 |
| 11 | N 22.31.912 | E 88.28.342 |
| 12 | N 22.31.187 | E 88.27.186 |
| 13 | N 22.33.425 | E 88.25.794 |
